# Synthesis of spiro-lactam hydrazones by clay catalysis: toxicity, antioxidant, hypolipidemic and *In silico* assessments

**DOI:** 10.1039/d6ra02313d

**Published:** 2026-05-05

**Authors:** Mohammed El Mesky, Hicham Zgueni, Ismail bouadid, Jarin Tasnim, Yassine Rhazi, Md Mehedi Hasan, Mohamed Tanghourte, Adil Qabouche, Mohammed Chalkha, Na'il Saleh, Driss Chebabe, El Houssine Mabrouk, Mohamed Eddouks

**Affiliations:** a Laboratory of Materials Engineering for the Environment and Natural Resources, Faculty of Sciences and Techniques, Moulay Ismail University of Meknes B.P 509, Boutalamine 52000 Errachidia Morocco m.elmesky@edu.umi.ac.ma; b Team of Ethnopharmacology and Pharmacognosy, Faculty of Sciences and Techniques Errachidia, Moulay Ismail University of Meknes Errachidia Morocco; c Department of applied Chemistry and Chemical Engineering, University of Rajshahi Rajshahi Bangladesh; d Laboratory of Engineering of Organometallic, Molecular Materials, and Environment (LIMOME), Faculty of Sciences Dhar EL Mahraz, Sidi Mohamed Ben Abdellah University P.O. Box 1796 (Atlas) Fez 30000 Morocco; e Chemistry Department, College of Science, United Arab Emirates University P.O.Box 15551 Al Ain United Arab Emirates

## Abstract

The development of sustainable synthesis methodologies for multifunctional bioactive molecules represents a critical challenge in green chemistry. Herein, we report the eco-efficient microwave-assisted synthesis of two novel spiro-lactam hydrazone derivatives (FHHA1, FHHA2) using abundant Moroccan Es-sifa natural clay as a separable and bifunctional heterogeneous catalyst. This three-step protocol achieves 86–87% overall yields in only 36 min, compared to 34–38% in 68 h under conventional heating representing an acceleration factor of 113. ^1^H/^13^C NMR, IR, and HRMS spectroscopy rigorously confirmed structures. Acute oral toxicity tests demonstrated the safety of both compounds, with a median lethal dose (LD_50_) between 2000 and 5000 mg kg^−1^. In Triton WR-1339-induced hyperlipidemic rats, FHHA1 (40 mg kg^−1^) significantly reduced LDL-c (*p* < 0.001), while FHHA2 (40 mg kg^−1^) decreased TC, TG, and LDL-c (*p* < 0.05, *p* < 0.05, *p* < 0.001). Both compounds exhibited complementary antihyperlipidemic effects, with FHHA1 showing superior LDL-c reduction. Furthermore, DPPH assays revealed dose-dependent antioxidant activity with IC_50_ values of 140.64 µg mL^−1^ and 181.91 µg mL^−1^. In addition to *in vivo* investigations, *in silico* studies were conducted, integrating molecular docking, molecular dynamics (MD) simulations, and Density Functional Theory (DFT) calculations, alongside comprehensive ADME analyses. This study establishes spiro-lactam hydrazones as a new class of environmentally friendly multifunctional agents, combining antioxidant and metabolic therapeutic potential through a base-free and sustainable catalytic process.

## Abstract 2

This study reports the synthesis of spiro-lactam hydrazones catalysed by Moroccan Es-sifa natural clay, with structural characterization, *in silico* studies and evaluation of biological activities.

## Graphical abstract

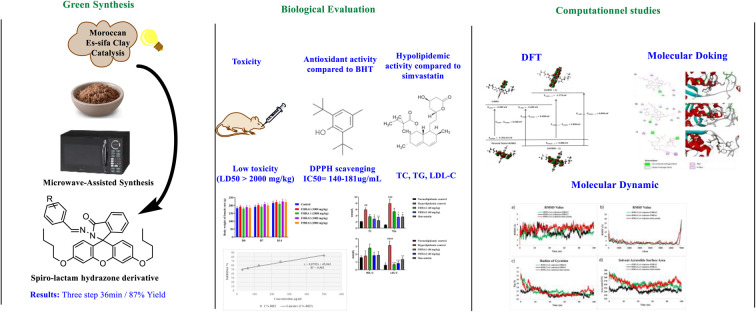

## 1. Introduction

Spiro-lactams represent an emerging class of rigid heterocycles in medicinal chemistry, characterized by a quaternary spiro center fused to γ- or δ-lactam rings.^1^ Beyond their fascinating architecture, these skeletons are frequently incorporated into bioactive molecules, notably as anticancer,^2^ antimicrobial^3^ and anti-inflammatory agents.^4^ When combined with the hydrazone moiety, a pharmacophore known for its broad biological activities including antioxidant,^5^ antihyperlipidemic,^6^ antimicrobial, and anticancer properties,^7^ these hybrid structures offer a promising platform for drug discovery.^8,9^

Various synthetic strategies have been developed to access these complex architectures. Metal-mediated approaches include zinc-catalyzed domino reactions,^10^ as well as copper- and palladium-catalyzed intramolecular cyclizations,^11^ cycloaddition methodologies encompass [1,3]-dipolar cycloadditions,^12^ and metal-free hetero-Diels–Alder reactions. Multicomponent strategies such as Passerini and Ugi reactions enable the rapid assembly of spiro-lactam frameworks.^1,13^ Additionally, condensation-based strategies, including imine formation and Knoevenagel–Michael cascade reactions, constitute efficient and practical approaches for the construction of these scaffolds.^14–16^ These motifs are of considerable importance in molecular design due to their structural rigidity and broad pharmacological relevance.^17,18^ However, the development of sustainable and environmentally benign synthetic methodologies remains a significant challenge.^19^

In this context, the principles of green chemistry encourage energy-efficient and waste-minimizing synthetic protocols. Microwave-assisted organic synthesis (MAOS) has emerged as a powerful technique, significantly reducing reaction times while improving yields and limiting side products.^20^ In the same line, natural mineral catalysts, particularly clays, are attracting growing interest. Their low cost, abundance and inherently eco-friendly nature make them ideal candidates. These heterogeneous systems offer concrete practical advantages, including easier separation and potential recyclability, thereby eliminating the need for toxic reagents.^21^ Furthermore, microwave irradiation is characterized by rapid and uniform heating, which improves the efficiency of catalytic processes and significantly reduces synthesis times.^22^ However, the combination of microwave irradiation and natural Moroccan clay Es-sifa for the synthesis of spiro lactam derivatives remains unexplored, offering a unique opportunity to exploit the specific mineralogical properties of this regional resource for complex chemical transformations.

This study reports the use of natural Moroccan clay Es-sifa as a bifunctional heterogeneous catalyst for the microwave-assisted synthesis of two novel spiro-lactam hydrazone derivatives (FHHA1 and FHHA2). The three-step protocol employs ethanol as a green solvent for the condensation steps, followed by solvent-free alkylation utilizing solely the intrinsic basicity of the raw clay representing a significant advancement over conventional base-mediated methods. Microwave irradiation demonstrated superior eco-efficiency compared to conventional heating. The antihyperlipidemic and antioxidant potential of FHHA1 and FHHA2 was evaluated through *in vivo* assays and acute toxicity studies. DFT calculations, molecular docking, MD and ADMET analyses provided insight into their binding interactions, structural stability and pharmacokinetic profiles. This work integrates green synthesis, natural resource valorisation and biological investigation, offering a sustainable approach for developing multifunctional bioactive agents.

## 2. Experimental

### 2.1. Materials and instrumentation

We used the Kofler bench to determine the melting points of the intermediate compounds and target compounds. CCM was used to monitor the progress of the reactions. All compounds synthesized in this manuscript were confirmed by spectroscopic methods, namely Bruker Advanced 300 WB NMR ^1^H NMR at 300 MHz and 600 MHz, as well as ^13^C NMR at 75 MHz and 125 MHz. The compounds were dissolved in DMSO-d_6_ for recording, taking tetramethylsilane (TMS) as an internal reference. We also used high-resolution mass spectroscopy to determine the exact mass of our compounds, using an UltiMate 3000 device (Thermo Scientific).

### 2.2. Preparation of Es-sifa clay

The clay used in this study, serving as a natural support and source of basicity for microwave-assisted reactions, was collected from Es-sifa, Morocco. Prior to use, the raw clay was washed several times with distilled water to remove soluble impurities, then dried at 80 °C for 24 h. The dried material was ground and sieved using ISO-compliant mesh sieves, and only particles with a size below 315 µm were retained for the synthesis experiments. The resulting clay was characterized by X-ray diffraction (XRD) and infrared (IR) spectroscopy to determine its mineralogical composition and surface functional groups, respectively.^23^

### 2.3. General synthetic procedures of FHHA1-2

#### 2.3.1. Synthesis of 2-amino-3′,6′-dihydroxyspiro[isoindolin-3-one-1,9′-xanthene] FH: using various methods- step 1

##### 2.3.1.1. Conventional method

First, 6.00 g of fluorescein is dissolved in 100 mL of anhydrous ethanol in a 250 mL three-necked round-bottom flask. Then, 6.0 mL of hydrazine is added slowly, taking approximately thirty minutes to do so. The mixture is subsequently heated to 80 °C and then maintained under reflux for about 24 h. The progress of the reaction is monitored by thin-layer chromatography (TLC). Once the reaction is complete, the solution is allowed to cool to room temperature, and the solvent is evaporated under reduced pressure. Next, 500 mL of water is added, which causes the formation of a solid. It is filtered under vacuum, washed three times with distilled water, and then dried. This yields fluorescein hydrazide, a light off-white solid, with a yield of approximately 76%.

##### 2.3.1.2. Microwave-assisted method

In a closed 250 mL reactor, 6 g of fluorescein (18.05 mM) and 6 mL (NH_2_NH_2_) are mixed with 10 mL of ethanol, with 10 g of a natural Moroccan clay (Es-sifa) as a heterogeneous catalyst support present. To activate the reaction, the mixture is then exposed to 400 W microwave irradiation for 9 min. After the reaction is complete, ethanol is added to reconstitute the organic phase. The natural support is then removed by simple filtration, and the solvent is evaporated under reduced pressure to isolate the crude product. Finally, the latter is purified by chromatography on silica gel columns using a 3 : 1 hexane/ethyl acetate mixture as the eluent affording the pure product in 96% yield as a light off-white solid.

#### 2.3.2. Synthesis of FHH1-2 using various methods- step 2

##### 2.3.2.1. Conventional method

In the second step, benzaldehyde derivatives (3-nitrobenzaldehyde and 4-nitrobenzaldehyde) were reacted with fluorescein hydrazine (FH) under reflux in methanol containing glacial acetic acid for 24 hours, yielding the fluorescein hydrazone derivatives FHH1-2 in moderate yield, (77.3% for FHH1 and 80.5% for FHH2).

##### 2.3.2.2. Microwave-assisted method

Mixtures of fluorescein hydrazide FH (0.348 g, 1 mmol),3-nitrobenzaldehyde (0.151 g, 1 mmol) or 4-nitrobenzaldehyde (0.151 g, 1 mmol), and 3 g of a Es-sifa clay in ethanol (EtOH, 5 mL) were kept in a closed reactor under microwave irradiation at 400 W for 12 min. Upon completion of the reaction, the reactor was allowed to cool to room temperature, and ethanol was added. The mixture was then filtered to remove the natural clay, and the solvent was evaporated under reduced pressure to isolate the crude product, which was purified by silica gel column chromatography using a 3 : 1 hexane/ethyl acetate mixture as eluent, affording the pure products in high yields (94.3% for FHH1 and 94.5% for FHH2).

#### 2.3.3. Synthesis of FHHA1-2 using various methods- step 3

##### 2.3.3.1. Conventional method

To a solution of the appropriate hydrazone derivative (FHH1 or FHH2, 1 mmol) in acetonitrile (10 mL) ware added triethylamine (TEA, 2 mmol, 0.28 mL) and 1-bromobutane (1.5 mmol, 0.16 mL). The reaction mixture was stirred at room temperature for 20 h. The progress of the reaction was monitored by thin-layer chromatography (TLC). After completion, the solvent was evaporated under reduced pressure. The residue was dissolved in ethyl acetate (30 mL) and washed with water (2 × 20 mL) and brine (20 mL). The organic layer was dried over anhydrous Na_2_SO_4_, filtered, and concentrated under reduced pressure. The crude product was purified by column chromatography on silica gel using a 4 : 1 hexane/ethyl acetate mixture as the eluent, affording the *O*-butyl hydrazone derivatives FHHA1-2 in moderate yields (58% for FHHA1 and 62% for FHHA2).

##### 2.3.3.2. Microwave-assisted method

A mixture of the appropriate hydrazone derivative FHH1 or FHH2 (1 mmol, 0.479 g), 1-bromobutane (1.5 mmol, 0.16 mL) and 3 g of a Es-sifa clay support was placed in a closed 50 mL reactor. The reaction mixture was then exposed to microwave irradiation at 200 W for 15 min. After completion of the reaction (monitored by TLC), the reactor was allowed to cool to room temperature. Ethanol (10 mL) was added to extract the product, and the mixture was filtered to remove the clay. The filtrate was concentrated under reduced pressure, and the residue was purified by column chromatography on silica gel using a 4 : 1 hexane/ethyl acetate mixture as the eluent, affording the *O*-butyl hydrazone derivatives FHHA1-2 in excellent yields (95% for FHHA1 and 96% for FHHA2).

### 2.4. Biological activities

#### 2.4.1. Experimental animals

Healthy albino adult male rats (Wistar strain) with a weight ranged between 180 and 220 g were housed under standard environmental conditions (23 ± 1 °C with 55 ± 5% humidity and a 12 h/12 h light/dark cycle) and maintained with free access to water and ad libitum standard laboratory diet. All animal procedures were performed in accordance with the Guidelines for the Care and Use of Laboratory Animals of Moulay Ismail University and were approved by the Animal Ethics Committee of the Faculty of Sciences and Techniques, Errachidia (Approval No. AREC-FSTE-12/2020).

#### 2.4.2. Acute oral toxicity

In accordance with OECD guidelines,^24^ an acute oral toxicity study of compounds FHHA1 and FHHA2 was conducted on normal, non-pregnant, and nulliparous rats. Twenty-one rats were randomly assigned to seven groups (I–VII), each consisting of three females. Group I received distilled water containing 1% DMSO and served as the control. Groups II, III, and IV were administered FHHA1 at doses of 1000, 2000, and 5000 mg kg^−1^, respectively, while Groups V, VI, and VII were administered FHHA2 at the same respective doses. All treatments were prepared in 1% DMSO. The animals were fasted overnight before administration.

Initially, one rat was fasted overnight and administered a starting dose of 1000 mg kg^−1^, and was observed for 48 hours. Based on the outcome, subsequent doses of 500, 1000, and 2000 mg kg^−1^ were administered. The animals were observed continuously during the first hours following administration, then hourly for the next 4 hours, and thereafter once daily for 14 days. Clinical signs of toxicity included changes in body weight, tremors, convulsions, excessive salivation, diarrhea, and alterations in mucous membranes, skin, and fur, which could potentially lead to death. Body weight was recorded on days 0, 7, and 14. The median lethal dose (LD_50_) was subsequently estimated.

#### 2.4.3. Anti-hyperlipidemic activity

Adult female rats weighing 180–220 g were used to assess the short-term effects of FHHA1 and FHHA2 at a dose of 40 mg kg^−1^ on hyperlipidemia induced by Triton WR-1339 (200 mg kg^−1^). The doses of 40 mg kg^−1^ of FHHA1 and FHHA2 were dissolved in DMSO at 1% (v/v). An intraperitoneal injection of Triton WR-1339 (Tyloxapol), dissolved in normal saline (pH 7.4), induced hyperlipidemia. Before induction, the animals were fasted overnight and then randomly assigned to five groups, each comprising six rats (*n* = 5):

Group 1 (normal control): received DMSO at 1% (v/v) orally 30 minutes before intraperitoneal injection of normal saline (pH 7.4). Group 2 (hyperlipidemia control): received 1% (v/v) orally 30 minutes before intraperitoneal injection of Triton WR-1339 (200 mg kg^−1^). Group 3: received FHHA1 (40 mg kg^−1^) 30 minutes before Triton WR-1339 injection. Group 4: received FHHA2 (40 mg kg^−1^) 30 minutes before Triton WR-1339 injection. Group 5 (standard group): received simvastatin (10 mg kg^−1^) 30 minutes before Triton WR-1339 injection. Twenty-four hours after treatment, the rats were anesthetized, and blood samples were collected from the retro-orbital sinus using heparinized capillaries. The samples were immediately centrifuged at 5000 rpm for 10 minutes, and the resulting serum was used for lipid profile analysis,^25^ such as TC, TG and HDL-c. LDL-c was calculated using the equation LDL-c = 0.7516  ×  (TC – HDL-c) as reported by de Cordova.^26^

#### 2.4.4. Antioxidant activity

The free radical scavenging activity of FHHA1 and FHHA2 was measured in terms of hydrogen donating or radical scavenging ability using the stable radical DPPH.^27^ A solution of DPPH was prepared by solubilizing 4 mg of DPPH in 100 ml of methanol. The solution was then placed in the dark for three hours. In glass tubes, a series of dilutions of the extract was prepared with a concentration of the stock solution of (5 mg/10 ml) in order to obtain the following concentrations (15.625, 31.25, 62.5, 125, 250 µg ml^−1^). For the dilution series of the positive control, butylhydroxytoluene (BHT) was prepared with a concentration of the stock solution of 5 mg/10 ml in order to obtain the following concentrations (31.25, 62.5, 125, 250, 500 µg ml^−1^). Then, 5 ml of the DPPH solution was introduced. After stirring, the tubes were placed in the dark at room temperature for 30 min. concerning the negative control, it contains a solution of DPPH and methanol, both with a volume of 2.5 ml. The reading is carried out by measuring the absorbance at 515 nm using a UV/vis spectrophotometer.^28^

Antioxidant activity of RSAE was estimated according to the following equation:(*I*%) = [*A*_control_ − *A*_sample_/*A*_control_] × 100where: (*I*%): percentage of the anti-radical activity; *A*_control_: absorbance of the control; *A*_sample_: absorbance of the sample.

The result was expressed by mean (of three separated measures) ± SEM.

#### 2.4.5. Statistical analysis

Data are expressed as mean ± SEM. Statistical analyses were performed using GraphPad Prism version 8 (GraphPad Software Inc., San Diego, CA, U.S.A). To determine statistical significance, one-way and two-way analyses of variance (ANOVA) were performed, followed by the Bonferroni test for multiple comparisons. Statistical significance was recognised as *p* < 0.05.

### 2.5. Computational method

#### 2.5.1. Protein retrieval, ligand preparation and molecular docking

The RCSB Protein Data Bank provided the crystal 3D structures of the target proteins, which included antibacterial, antioxidant, and anti-cancer targets.^29^ The water molecules and heteroatoms were removed from the protein using Biovia Discovery Studio.^30^ The protein was then loaded into Swiss-Pdb-Viewer for energy minimization and model residual breakage by Modeller in Chimera-X 1.10.1.^31,32^ The ligands were aggregated into a single SDF file using Open Babel, then converted to pdbqt format in PyRx, and their energy was decreased using PyRx's built-in force fields.^32,33^ Molecular docking with Autodock vina in PyRx was employed to acquire information about the interactions.^34,35^ Docking was performed using pdb structures provided from the RCSB Protein Data Bank. For docking purposes, the proteins were prepared by removing water molecules and heteroatoms, as described previously. The cleaned structures were then loaded into PyRx and converted into macromolecules for docking. Ligands, initially in Babel format, were converted to SDF format and subjected to energy minimization using the Ghemical force field. To achieve optimal binding affinity and interactions, each protein was oriented to encompass its corresponding active site. Docking outcomes were evaluated based on the binding scores of each protein–ligand pair. The resulting binding poses were visualized using PyMol, while hydrogen bonding and non-bonded interactions were analysed with Discovery Studio.^36^

#### 2.5.2. DFT calculation

Density Functional Theory (DFT) provides insight into the intrinsic electronic characteristics of compounds, complementing docking studies by validating a compound's drug-likeness at the quantum level. Although molecular docking predicts binding poses and affinities, it may not fully capture electronic contributions to binding interactions.^37^ In contrast, DFT calculates the precise calculation of key electronic parameters, including frontier molecular orbital (FMO) energies, their energy gap (Δ*E*), molecular electrostatic potential (MEP), dipole moment, polarizability, and other chemical reactivity parameters. These parameters give us insight into electron-donating and accepting capacities, molecular softness or hardness, and the likelihood of hydrogen bonding and electrostatic interactions.^38,39^

For the evaluation of the electronic properties by DFT, the B3LYP exchange–correlation functional combined with the 6-31G(d,p) basis set was used to due to its reliable balance between computational efficiency and accuracy.^40^ Integrating DFT-derived electronic descriptors with docking results, the drug-like properties of ligands can be explained with further validation. This combined approach strengthens predictions regarding binding stability, interaction specificity, and the overall reliability of *in silico* drug discovery strategies.

In the study, DFT calculations were carried out using Gaussian software.^41,42^ Initial energy minimization was performed using Gabedit.^43^ All computational calculations were carried out with B3LYP/6-31G(d,p) level of theory.^44^ Geometry optimization was performed to obtain the most stable molecular conformation, followed by evaluation of frontier molecular orbitals (HOMO–LUMO), the corresponding energy gap, molecular electrostatic potential (ESP) surface, and additional electronic characterization parameters.^45^

The global reactivity parameters were derived from the calculated HOMO and LUMO energies according to the following relations:*A* = −*E*_LUMO_*I* = −*E*_HOMO_Δ*E* = *E*_LUMO_ − *E*_HOMO_*µ* = −(*I* + *A*)/2*η* = (*I* − *A*)/2*S* = 1/*η**ω* = *χ*^2^/2*η**χ* = (*I* + *A*)/2

#### 2.5.3. Molecular dynamic simulation

Molecular dynamics (MD) simulations were performed to evaluate the time-dependent conformational stability of the protein–ligand complexes and to further investigate the stability of the selected therapeutic candidates.^46^ All simulations were carried out using GROMACS 2022 in a Linux Ubuntu environment, with separate simulations conducted for the apo protein and the corresponding protein–ligand complexes. The overall trajectory time for each system was simulated of 100 ns.^47^ By utilizing the CHARMM36 force field and the TIP3P water model to explain the manner in which the molecules in the system interact with each other. The simulations were performed under periodic boundary conditions, with the temperature maintained at 300 K and the pressure at 1 bar. Initially, energy minimization was carried out to remove unfavorable steric clashes and structural distortions in the system.^48^ This step was followed by equilibration under constant volume and temperature (NVT) and constant pressure and temperature (NPT) ensembles with position restraints applied to stabilize the system.^49^ Harmonic restraints were used during the equilibration phase to maintain the structural integrity of the solute molecules. After equilibration, the protein–ligand complexes were subjected to a production MD simulation consisting of 50, 000, 000 steps. Subsequently that, the resulting trajectories were analyzed at to obtain a corresponding number of structural and dynamic parameters, such as root mean square deviation (RMSD), root mean square fluctuation (RMSF), radius of gyration (*R*_g_), solvent-accessible surface area (SASA), and hydrogen-bonding patterns throughout the simulation.

#### 2.5.4. ADME and toxicity analysis

The pharmacokinetic (PK) and drug-likeness profiles of the docked ligands were also predicted using SwissADME, and the ADMETlab 3.0 web server.^50,51^ These were used to explore the ADMET profiles of each compound, including Lipinski's rule of five and other descriptors. Toxicity profiles, LD_50_ values, and potential toxicological hazards of the selected ligands were assessed using the Protox 3.0 server.^52^ The collective outcomes provided clues about the pharmacologic potential and safety of the tested compounds.

## 3. Results and discussion

### 3.1. Chemistry and characterization

This strategy introduces several major methodological innovations compared with the methods described in the literature. The pivotal use of natural Moroccan Es-sifa clay as a bifunctional heterogeneous catalyst enables the *O*-alkylation step to be carried out under microwave irradiation without the addition of any external synthetic base, which represents a significant advance over conventional protocols. This dual role of the clay, acting as both a solid support and a natural reservoir of basicity, highlights its effectiveness in the development of highly sustainable and environmentally friendly processes.

**Scheme 1:**
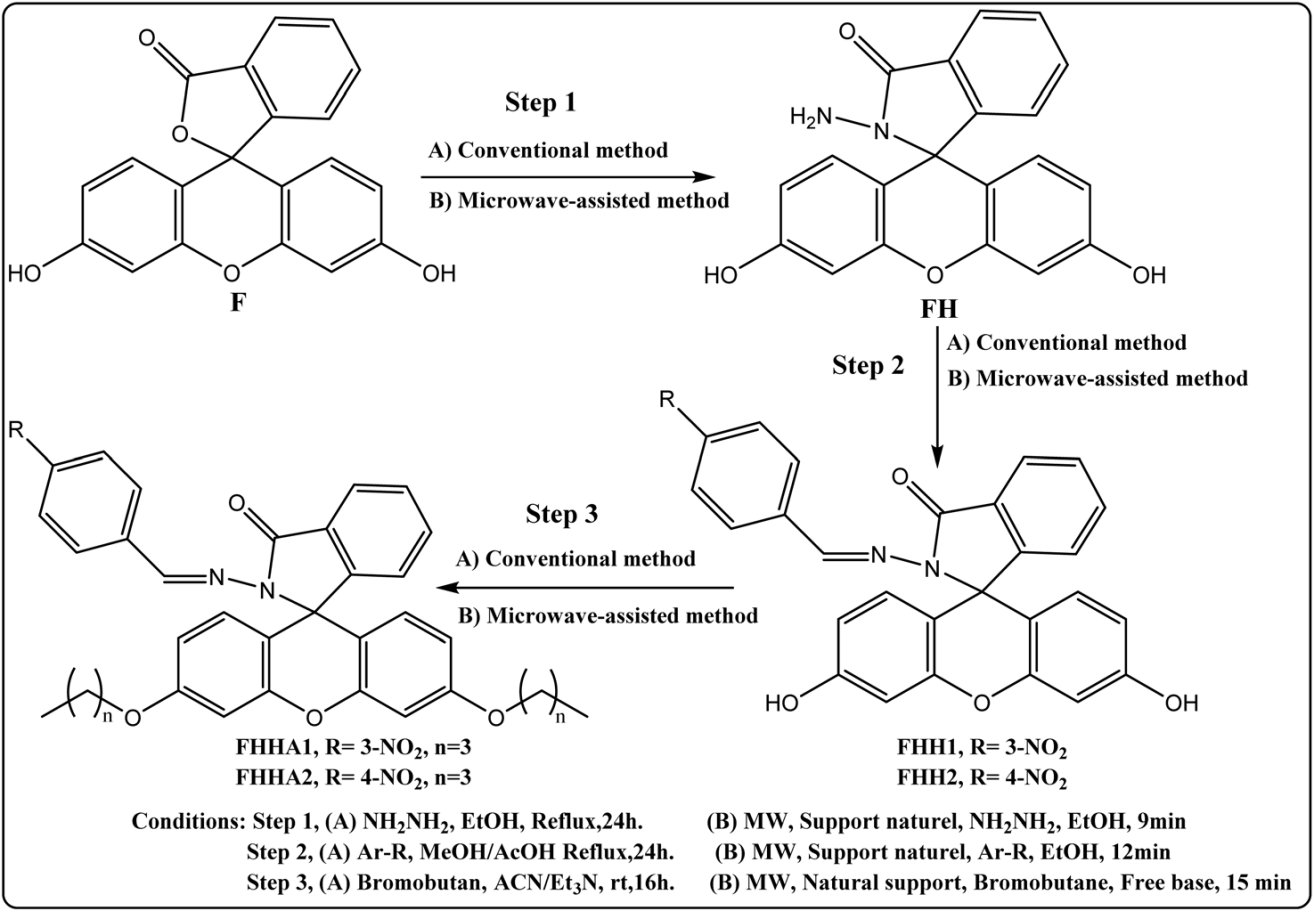
Synthetic route for the preparation of spiro-lactam hydrazone derivatives FHHA1 and FHHA2.

The synthetic route was initiated using fluorescein (FF) in lactone form. The hydrazide derivative (FH) was synthesized using two comparative approaches, a conventional method,^53^ and a microwave-assisted method using the natural support as a catalyst. This intermediate was then condensed with nitrobenzaldehyde derivatives to form the hydrazone derivatives FHH1–2, using both a conventional method,^54^ and a microwave-assisted route using our locally prepared clay catalyst to yield the hydrazone derivatives FHH1–2 subsequently, the critical *O*-alkylation step was performed using bromobutane. While conventional conditions require triethylamine (Et_3_N) as a hazardous organic base, the microwave-assisted reaction proceeds efficiently in a base-free environment, driven solely by the intrinsic basicity of the Es-sifa clay. This leads to the formation of two novel functionalized molecules, FHHA1 and FHHA2 (Scheme 1). Comparative studies clearly show that the synergy between microwave irradiation and natural catalysis significantly improves the efficiency of all synthesis steps.

**Fig. 1:**
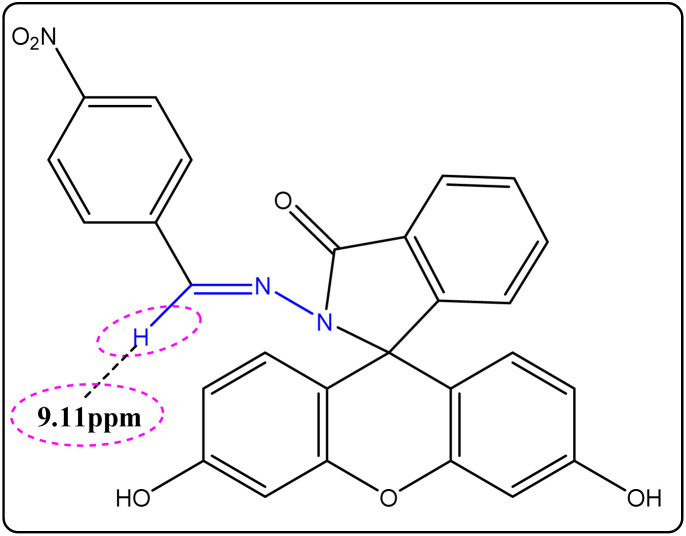
^1^H NMR chemical shift of the azomethine (CH=N) proton in the hydrazone compound FHH2.

The hydrazone moiety is responsible for the distinctive signals seen in the ^1^H NMR spectra of the synthesized compounds FHH1 and FHH2. Specifically, a highly unshielded environment is indicated by the azomethine proton (N=CH), which shows up as a signal at about 9 ppm. According to the literature, which generally favors the *syn*-periplanar (sp) conformer because of its lower steric hindrance, this high value indicates the predominance of this conformer (Fig. 1).^55^

#### 3.1.1. Spectroscopic data (NMR, IR and HRMS)

All the products obtained were purified by column chromatography and then characterised by various spectroscopic methods, including NMR (^1^H and ^13^C), and IR before being confirmed by high-resolution mass spectrometry. The samples were prepared in DMSO-d_6_ for NMR analysis.

For example, the ^1^H NMR spectrum of compound FHHA2, recorded in DMSO-d6, shows a triplet at 0.87 ppm corresponding to the six protons of the two methyl groups (C–CH_3_), as well as a broad signal at 1.38 ppm attributed to the four protons of the two methylene groups (–CH_2_–CH_3_). Another signal at 1.63 ppm is related to the four protons of the two methylene groups O–CH_2_–CH_2_. In addition, a triplet at 3.95 ppm corresponds to the six protons of the two methylene groups Ar–O–CH_2_– attached to the aromatic ring. The presence of the hydrazone (CH=N) function is confirmed by a singlet at around 9.32 ppm, while the signals between 6.5 and 8 ppm correspond to the aromatic protons.

In the ^13^C NMR spectrum of the same compound, a signal at 14.15 ppm is attributed to the two carbons of the methyl groups (–CH_3_) in the aliphatic chain. Another signal at 19.21 ppm corresponds to the methylene carbons (–CH_2_–) linked to the terminal methyl groups. A peak characteristic of the (O–CH_2_–) groups is observed at 68.12 ppm, while the spiro carbon, specific to fluorescein hydrazone, appears at 65.76 ppm.

The IR spectroscopy shows an intense band at 1702 cm^−1^ (*ν* C=O lactam/ester), 2989–2872 cm^−1^ (*ν*CH_2_, *ν*CH_3_), and 1301–1014 cm^−1^ (*ν*C–O–C, *ν*C–N). Bands at 3089–3020 cm^−1^ and 873–684 cm^−1^ confirm aromatic C–H stretching and bending vibrations.

HRMS confirmed the expected molecular mass, with the detection of the pseudomolecular ion [M + H]^+^ at *m*/*z* 592.23609, thus validating the structure of compound FHHA2 as well as that of the other derivative.

#### 3.1.2. Comparison between microwave (MW) and conventional methods

Microwave (MW) heating offers several advantages over conventional heating, owing to its direct interaction with matter at the molecular level, which enables rapid and volumetric heating while minimizing heat losses through conduction and convection. This makes MW irradiation particularly efficient in organic synthesis and heterogeneous catalysis, especially through selective heating of active sites.^56–58^ The rapid and uniform temperature rise under microwave irradiation minimizes side reactions and thermal degradation of reactants or products, while accelerating the condensation and alkylation steps on the clay surface. In addition, the selective heating of the clay catalyst leads to a higher local temperature at the active sites,^59^ thereby enhancing the reaction rate and favoring the formation of the desired spiro-lactam hydrazone derivatives.

The experimental results (Table 1 and 2) confirm these advantages, showing significantly higher yields (86.0% and 87.1%) compared to the conventional method (34.1% and 37.9%), along with a drastic reduction in reaction time (from 68 h to 36 min). These improvements are also reflected in the higher values of reaction efficiency and overall yield efficiency, confirming the superiority of the microwave-assisted approach in terms of efficiency and process sustainability.

**Table 1:** Comparison of time and YE for three synthesis steps: conventional *vs.* microwave methods

Compound	Step	Microwave method			Conventional method		
		Time (min)	Yields (%)	YE	Time (min)	Yields (%)	YE
FH	1	9	96	10.7	1440	76	0.05
FHH1	2	12	94.3	7.9	1440	77.3	0.05
FHH2	2	12	94.5	7.9	1440	80.5	0.06
FHHA1	3	15	95	6.3	1200	58	0.05
FHHA2	3	15	96	6.4	1200	62	0.05

**Table 2:** Comparison of total reaction time and YEG between conventional and microwave methods

Compound	Microwave method			Conventional method			Mp (°C)
	Time (min)	Global yields (%)	YEG	Time (min)	Yields (%)	YEG	
FHHA1	36	86.0	2.4	4080	34.1	0.008	129–131
FHHA2	36	87.1	2.4	4080	37.9	0.009	122–124

The efficiency parameter (YE) and YEG was calculated using the following equation, as previously reported in the literature.^60^
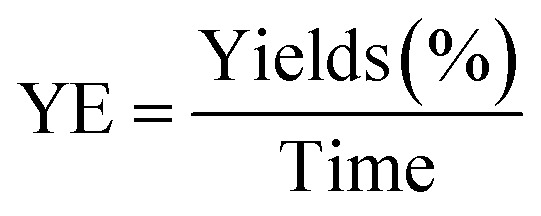
And YE is Yield EconomicGlobal Yields(%) = Yields(steep1) × Yields(steep2) × Yields(steep3)global Time(min) = Time(steep1) + Time(steep2) + Time(steep3)
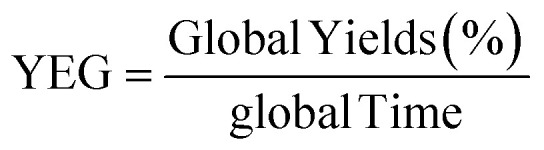
In addition, YEG is Yield Economic Global.

### 3.2. Biological activities

#### 3.2.1. Acute toxicity

**Fig. 2:**
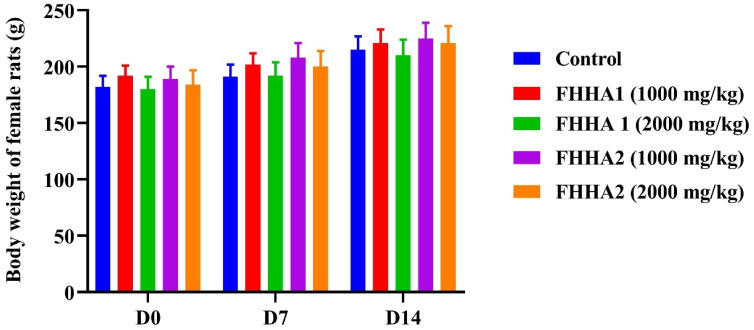
Body weight changes in female rats from the control group and those treated with FHHA1 and FHHA2 at doses of 1000 and 2000 mg kg^−1^ during the acute toxicity study. Values are expressed as mean ± SEM (*n* = 3).

The acute toxicity study of FHHA1 and FHHA2 compounds in rats revealed no mortality or observable signs of toxicity at doses of 1000 and 2000 mg kg^−1^. Furthermore, food and water intake remained unchanged, and no signs of aggression, salivation, contortions, or piloerection were observed in treated animals. In contrast, administration of the 5000 mg kg^−1^ dose induced general weakness and resulted in the death of all animals within 24 hours after administration. During the 14 day observation period, the animals maintained a normal health status, with steady body weight gain (Fig. 2). No statistically significant differences in body weight were observed between the groups treated with FHHA1 and FHHA2 at doses of 1000 and 2000 mg kg^−1^ and the control group (*p* > 0.05). These findings allow us to estimate that the median lethal dose (LD_50_) lies between 2000 and 5000 mg kg^−1^ (Fig. 2), indicating a relatively low level of acute toxicity.

The toxicological evaluation of FHHA1 and FHHA2 revealed a DL > 1000 mg kg^−1^, confirming the innocuity of these spiro-lactam hydrazones. The biocompatibility of the structural motif is validated by the absence of any signs of toxicity.

#### 3.2.2. Antihyperlipidemic effects

Administration of Triton WR-1339 (Tyloxapol) induced a significant increase in plasma levels of total cholesterol (TC), triglycerides (TG), and LDL cholesterol (LDL-c) (*p* < 0.01, *p* < 0.001, *p* < 0.0001, respectively) compared to the normolipidemic control group. In contrast, plasma HDL-c levels were not significantly altered compared to the normolipidemic control group.

**Fig. 3:**
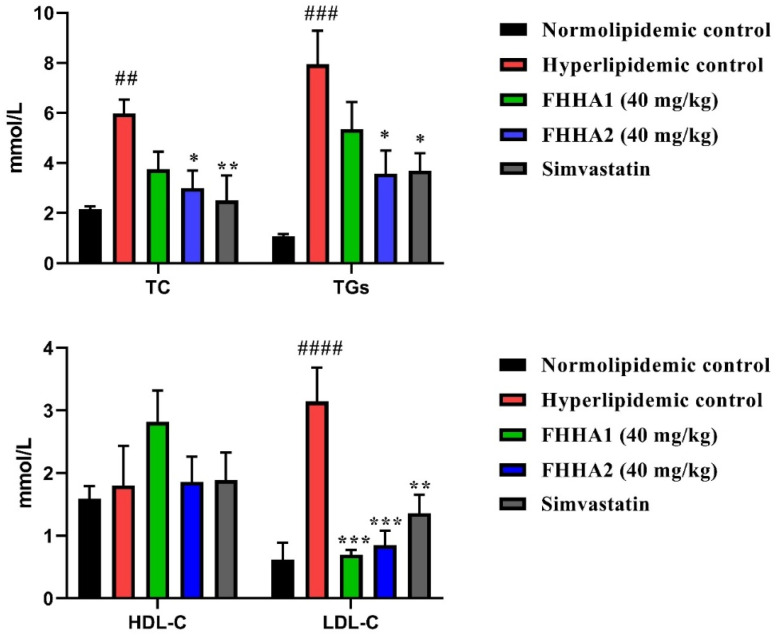
Effect of FHHA1 (40 mg kg^−1^) and FHHA2 (40 mg kg^−1^) on plasma lipid profile (TC, TG, HDL-c and LDL-c) in Triton WR-1339-induced hyperlipidemic rats. Data are reported as mean ± SEM. for 5 animals per group. **p < 0.01, ***p < 0.001, and ****p < 0.0001 compared to normolipidemic group, #*p* < 0.05, ##*p* < 0.01, ###*p* < 0.001 and ####*p* < 0.0001 compared to Triton WR-1339 group.

Treatment of hyperlipidemic rats with the compound FHHA1 at a dose of 40 mg kg^−1^ resulted in a significant decrease in plasma LDL-c levels (*p* < 0.001) compared to the hyperlipidemic control group. While administration of FHHA2 at the same dose (40 mg kg^−1^) induced a significant reduction in plasma levels of TC, TG, and LDL-c (*p* < 0.05, *p* < 0.05, *p* < 0.001, respectively), simvastatin also resulted in a significant decrease in plasma levels of TC, TG, and LDL-c (*p* < 0.01, *p* < 0.05, *p* < 0.01, respectively) (Fig. 3).

FHHA2 (40 mg kg^−1^) significantly reduced TC, TG, and LDL-c levels, while FHHA1 selectively lowered LDL-c without affecting other parameters. This structure-activity relationship may arise from the nitro (NO_2_) group in FHHA2, which enhances electron conjugation with the hydrazone moiety and optimizes interactions with nuclear receptors regulating lipid metabolism; in contrast, FHHA1's substitution limits its action to LDL-c catabolism.

Furthermore, the observed antihyperlipidemic activity may be partially associated with the antioxidant properties of the compounds, as oxidative stress plays a key role in lipid metabolism disorders.^61,62^ Therefore, the antioxidant activity of FHHA1 and FHHA2 was also investigated.

#### 3.2.3. Antioxidant activity

**Fig. 4:**
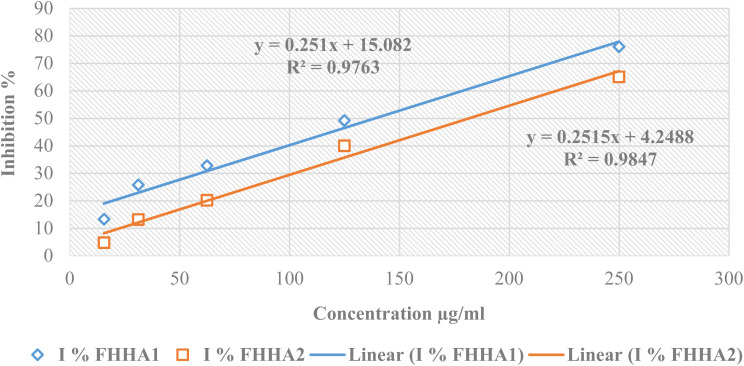
Percentage of inhibition of DPPH as a function of the concentrations of FHHA1 and FHHA2.

**Fig. 5:**
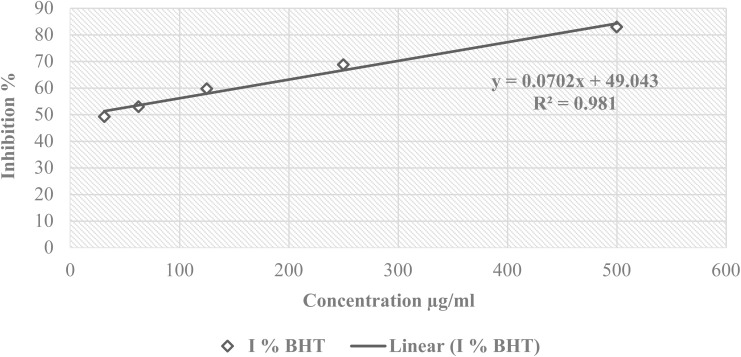
Percentage of inhibition of DPPH as a function of the concentrations of BHT: butylhydroxytoluene.

Different concentrations of FHHA1 and FHHA2 (15.625, 31.25, 62.50, 125 and 250 µg mL^−1^) exhibited dose-dependent antioxidant activity. The percentages of inhibition (*I*%) obtained for FHHA1 were 13.30%, 25.77%, 32.77%, 49.18% and 75.98%, respectively, while those obtained for FHHA2 were 4.76%, 13.16%, 20.16%, 39.97% and 65.02% (Fig. 4). For the different concentrations of BHT (31.25, 62.50, 125, 250, and 500 µg mL^−1^), used as the reference antioxidant, the observed inhibition percentages were 49.17%, 52.89%, 59.67%, 68.66%, and 82.87%, respectively (Fig. 5). Linear regression analysis was used to determine the IC_50_ inhibitory concentration values. The IC_50_ values for FHHA1 and FHHA2 were 140.64 µg mL^−1^ and 181.91 µg mL^−1^, respectively, while BHT had an IC_50_ of 13.63 µg ml^−1^.

The antioxidant activity of FHHA1 and FHHA2 was found to be lower than that of the reference compound BHT. This can be attributed to the absence of free phenolic hydroxyl groups in their structures, which are essential for efficient hydrogen atom donation *via* hydrogen atom transfer (HAT) or single electron transfer (SET) mechanisms.^63,64^ In contrast, BHT is a well-known phenolic antioxidant with high radical scavenging efficiency due to its ability to donate hydrogen atoms and form stable phenoxyl radicals.^65^ Furthermore, the bulky and rigid structure of the synthesized compounds may introduce steric hindrance, limiting their interaction with free radicals and reducing their scavenging efficiency.^66^ Despite this, FHHA1 exhibited moderate antioxidant activity, suggesting that structural modification could further enhance its performance.

### 3.3. Computational studies

The current computational study was performed to thoroughly assess the antihyperlipidemic activity of the synthesized spiro-lactam derivatives FHHA1 and FHHA2 by integrating an *in silico* approach comprising molecular docking, MD simulations, DFT and ADME-Tox profiling. To ensure a consistent comparative framework, simvastatin, a common HMG-CoA reductase inhibitor, was used as the reference drug.^67^ This integrated computational pipeline provides deep mechanistic insights into the binding stability, electronic properties, and pharmacokinetic behavior of the investigated complexes.^68^

#### 3.3.1. Molecular docking study

**Fig. 6:**
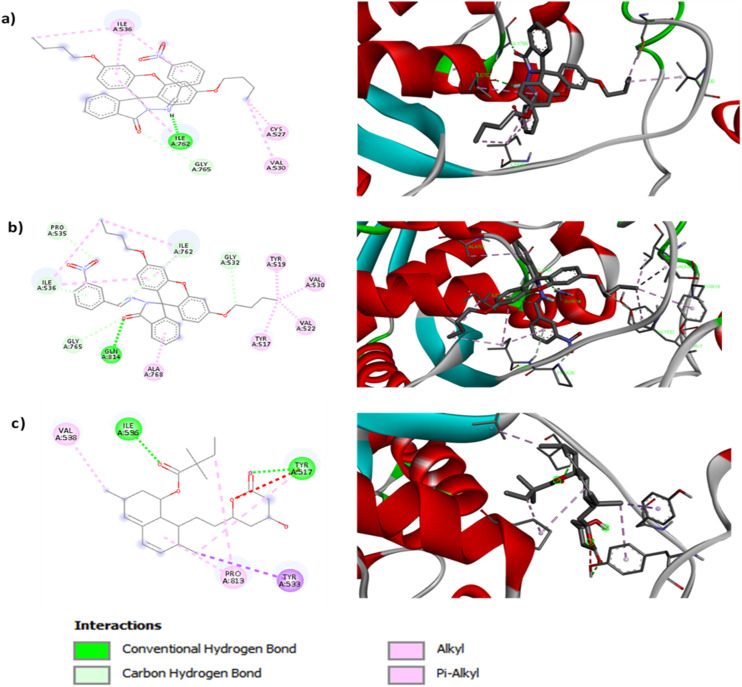
2d and 3d interaction of (a) FHHA1 (b) FHHA2 (c) simvastatin.

To better understand the molecular basis underlying the experimentally demonstrated superiority of FHHA2 in reducing total cholesterol (TC), triglycerides (TG) and LDL-c (*p* < 0.05–0.001; Fig. 3), molecular docking simulations were performed against HMG-CoA reductase (the validated target of simvastatin) and compared with the reference drug.^69^ The binding affinity of simvastatin was relatively low (−7.7 kcal mol^−1^), whereas those of FHHA1 and FHHA2 were −8.1 and −8.5 kcal mol^−1^, respectively. This finding indicates that the two synthetic spiro-lactam derivatives were more capable of binding to the catalytic region of the target enzyme. Fig. 6 depicts the 2D & 3D interactions. Among them, FHHA2 exhibited a slightly higher binding affinity and superior biological potency, making it a more favorable candidate than simvastatin.^70^ This enhanced activity correlates with its broader antihyperlipidemic effect observed in Triton WR-1339-induced hyperlipidemic rats (Fig. 3), where FHHA2 significantly reduced TC, TG, and LDL-c levels, whereas FHHA1 showed a selective effect primarily on LDL-c.

Detailed analysis of the protein-ligand interactions revealed that the *para*-nitro substitution in FHHA2 enables a significantly more diversified interaction network compared to the *meta*-nitro in FHHA1, directly explaining their differential pharmacological profiles. While FHHA1 forms predominantly hydrophobic interactions with Ile536, Ile762, Val522, Val530 and Ala768, The recurrent association of the ILE536 and ILE762 indicate a conserved and steady accommodation in the hydrophobic core of the binding cavity.^71^ Furthermore, CYS527 interaction can also play a role in weak polar stabilization, which is dependent on the geometry of the ligand. FHHA2 establishes additional polar contacts with Tyr517 and Gln814 through hydrogen bonds and π-based interactions (Fig. 6). The conformational orientation of the pocket in the presence of PRO535 can also be affected. These supplementary polar interactions, geometrically enabled by the nitro group of *para*-position, likely account for the comprehensive lipid-lowering activity of FHHA2 (TC↓, TG↓, LDL-c↓) compared to the selective LDL-c reduction by FHHA1. The *meta*-position in FHHA1 constrains the nitro group orientation, limiting its ability to engage these critical polar residues and restricting its activity to LDL-c modulation.

In contrast, simvastatin engages conventional hydrogen bonds with Tyr517 and Ile536 plus hydrophobic interactions with Tyr533, Val538 and Pro813. The limited diversity of simvastatin interactions correlates with its established pharmacological profile, whereas the enhanced interaction networks of synthetic spiro-lactam derivatives particularly FHHA2 explain their superior experimental efficacy.

These findings were consistent with the experimental work as the stronger binding affinity and larger interaction network of FHHA2 indicates a more stable accommodation in the active site, which makes it superior to the pharmacological activity of FHHA1. Capacity of forming hydrophobic and polar interactions must increase its inhibitory potential, and the relative simplicity of the interaction pattern of FHHA1 may serve as a reason behind its comparative low biological activity.^71^ Altogether, the outcome of the docking shows that the *meta vs. para* position of nitro group affects the degree of antihyperlipidemic action.

#### 3.3.2. Molecular reactivity analysis (DFT study)

**Fig. 7:**
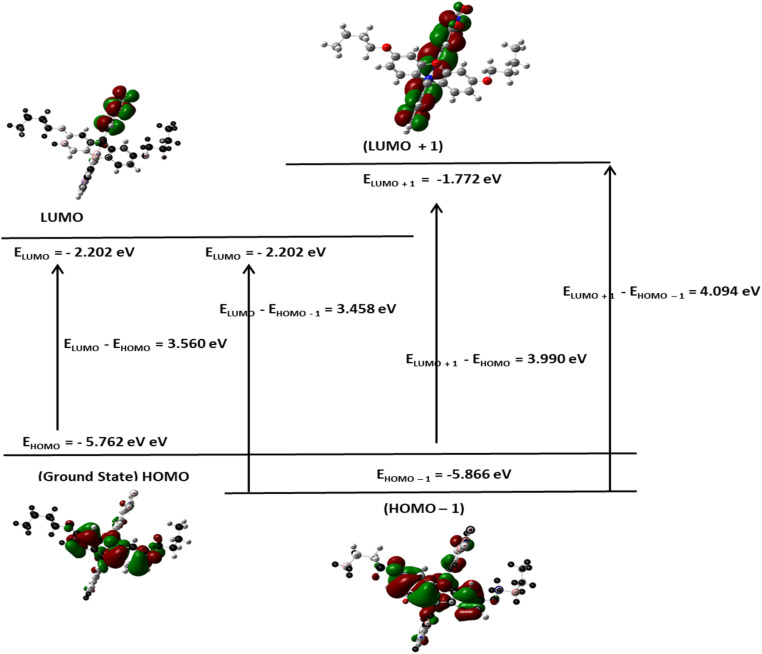
FMO analysis of FHHA1.

**Fig. 8:**
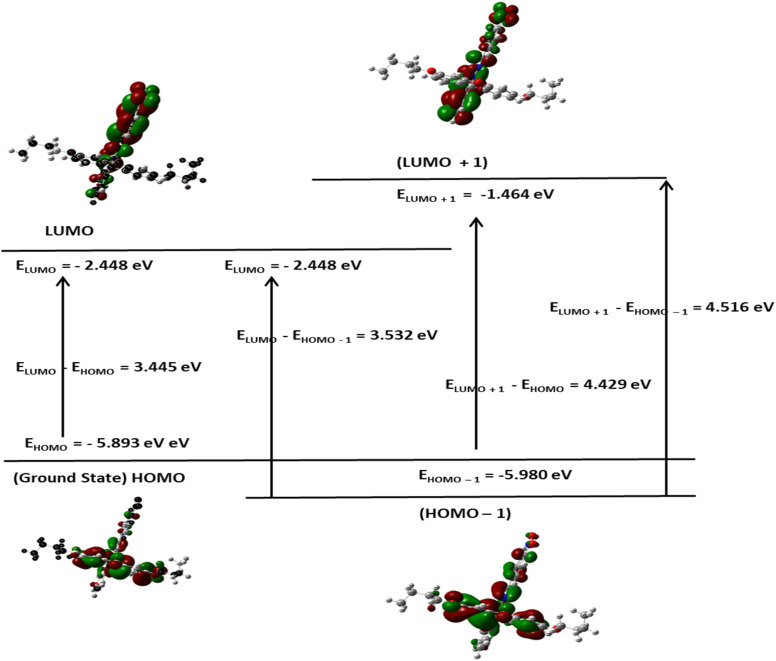
FMO analysis of FHHA2.

**Fig. 9:**
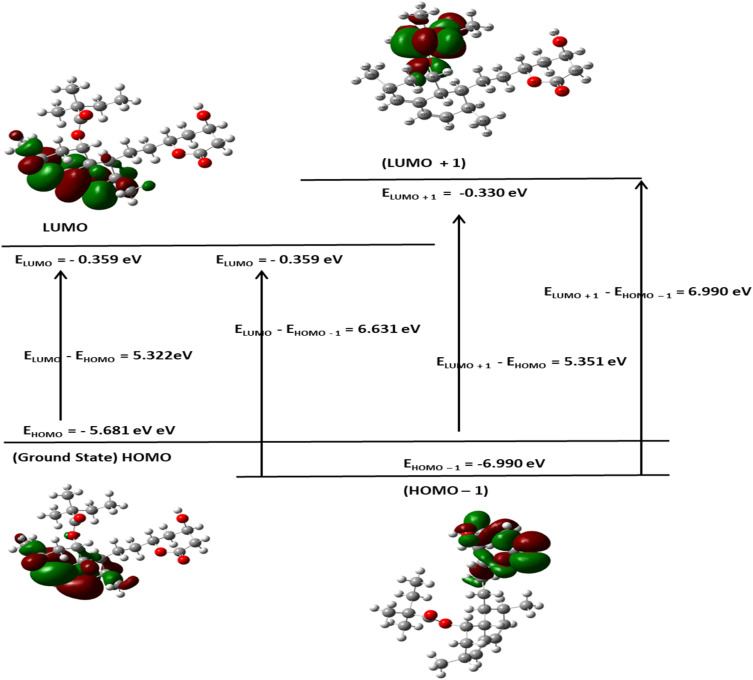
FMO analysis of simvastatin.

Although the FHHA2 antioxidant ability was better explained by docking, DFT calculations were used to justify the inverse relationship between the antioxidant ability of both the derivatives where FHHA1 (IC_50_ = 140.64 µg mL^−1^) scavenges DPPH radical more than FHHA2 (IC_50_ = 181.91 µg mL^−1^). The electronic properties considered by the DFT analysis were chemical reactivity and interaction potential, the frontier molecular orbital parameters calculated were presented as Fig. 7, 8 and 9.^72^

The HOMO energy of FHHA1 was −5.762 eV and the LUMO energy was −2.202 eV, which gave 3.560 eV as the energy gap (DE). Likewise, FHHA2 gave *E*_HOMO_ = −5.893 eV and *E*_LUMO_ = −2.448 eV, with a slightly lower DE of 3.445 eV. Simvastatin, on the contrary, exhibited an energy gap that was much larger (5.322 eV; *E*_HOMO_ = −5.681 eV; *E*_LUMO_ = −0.359 eV). Indicating enhanced electronic flexibility. The reduced DE values of synthetic spiro-lactam derivatives represent increased electronic reactivity and charge transfer ability over simvastatin, which has a larger energy gap, representing higher kinetic stability, but lower re-configurability during molecular recognition.^73^ Critically, examination of the HOMO electron density distribution (Fig. 7–9) revealed that the *meta*-nitro position in FHHA1 preserves higher electron density on the azomethine imine (CH=N) compared to the *para*-nitro in FHHA2. This higher electron density facilitates proton transfer to the DPPH radical, explaining its superior antioxidant potency observed experimentally.

The ionization potential (IP) values are as follows: FHHA2 (5.893 eV) > FHHA1 (5.762 eV) > simvastatin (5.681 eV), implicating similar donating tendencies of the compounds on electrons (Table 3). Nonetheless, electron affinity (*E*_A_) values are quite different where FHHA2 (2.448 eV) and FHHA1 (2.202 eV) are quite higher than that of simvastatin (0.359 eV). It shows that spiro-lactam derivatives have greater electron acceptance ability specially FHHA2 which can be useful in increasing the electrostatic stabilization of the enzyme active site.

**Table 3:** The global reactivity parameters

Parameters	FHHA1	FHHA2	Simvastatin
*E*_HOMO (eV)	−5.762	−5.893	−5.681
*E*_LUMO (eV)	−2.202	−2.448	−0.359
Δ*E* (energy gap, eV)	3.560	3.445	5.322
IP (ionization potential, eV)	5.762	5.893	5.681
EA (electron affinity, eV)	2.202	2.448	0.359
*µ* (chemical potential, eV)	−3.982	−4.171	−3.020
*η* (hardness, eV)	1.780	1.723	2.661
*S* (softness, eV^−1^)	0.281	0.290	0.188
*ω* (electrophilicity index, eV)	4.450	5.050	1.710
*χ* (electronegativity, eV)	3.982	4.171	3.020

The observation can be further supported by the chemical hardness (*e*) and softness (*S*). The hardest (2.661 eV) and the softest (0.188 eV^−1^) simvastatin molecules have the highest and the lowest hardness and softness respectively, and this implies that its electronic structure is relatively rigid. FHHA2 (*e* = 1.723 eV; *S* = 0.290 eV^−1^) and FHHA1 (*e* = 1.780 eV; *S* = 0.281 eV^−1^) on the other hand exhibit greater softness, which is an indication of improved polarizability and flexibility in the biological surroundings.^74^ The fact that FHHA2 (4.171 eV) and FHHA1 (3.982 eV) have higher values of electronegativity than simvastatin (3.020 eV) is another indication that they have better electron-attracting powers.

Conversely, the *para*-nitro in FHHA2 enables extended π-conjugation, delocalizing electron density away from the azomethine and stabilizing the hydrazone tautomer. While this conjugation optimizes electrophilicity for enzyme binding (consistent with FHHA2's superior docking score and antihyperlipidemic activity), it simultaneously reduces hydrogen-donating ability, directly accounting for the higher IC_50_ observed experimentally.

The electrophilicity index (*ω*) follows the order: FHHA2 (5.05 eV) > FHHA1 (4.45 eV) >> simvastatin (1.71 eV). This reveals the fundamental electronic trade-off: higher electrophilicity favors enzyme inhibition (FHHA2's broad antihyperlipidemic activity), while moderated electrophilicity preserves radical scavenging capacity (FHHA1's superior antioxidant activity).

The results of DFT are consistent with the experimental data, which suggests that the reduced energy difference, increased electrophilicity and increased softness of spiro-lactam derivatives increase the electronic flexibility and the potential of the interactions. FHHA2 had the lowest energy gap and highest electrophilicity, indicating higher potential of charge transfer and stabilization in the enzymatic binding site. These electronic characteristics assist the docking results and assist in the explanation of the superior pharmacological activity found in FHHA2 favors antihyperlipidemic activity.

#### 3.3.3. MEP and NCI analyses

**Fig. 10:**
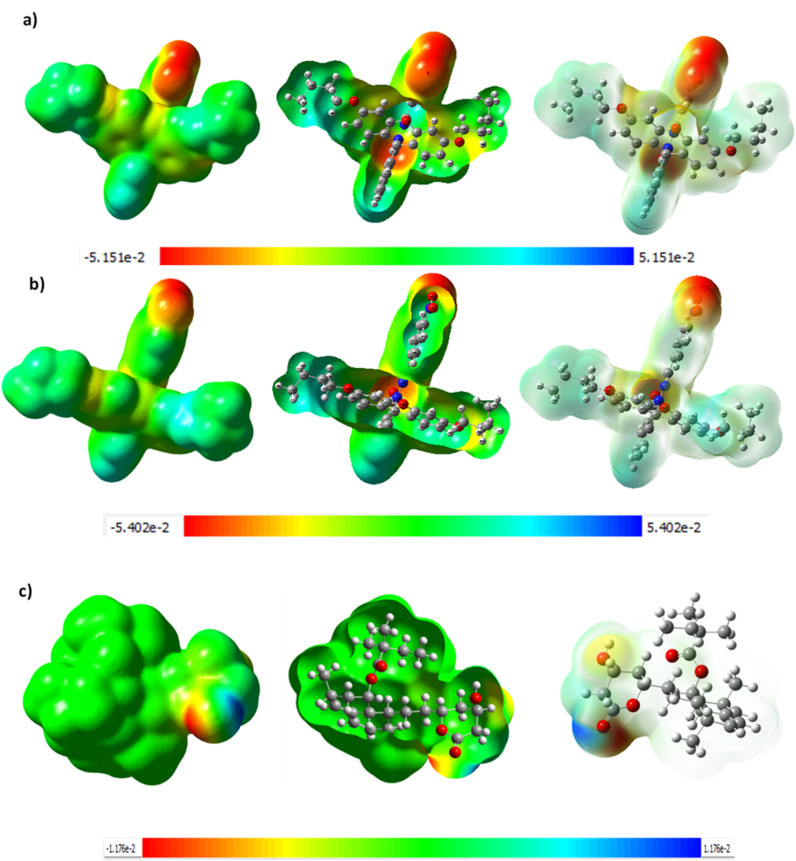
MEP analysis of (a) FHHA1 (b) FHHA2 (c) simvastatin.

Molecular electrostatic potential surface of FHHA1 and FHHA2 shown in Fig. 10 indicate that these two proteins have clear electron-rich and electron-deficient regions, which determine their interaction behavior. The red areas concentrated on oxygen functional groups reveal the presence of high hydrogen bond acceptance capacity and the blue areas could have donor sites, increases the affinity to the amphiphilic enzyme pockets, which are related to lipid metabolism. The spiro-lactam derivatives exhibit more negative electrostatic potential than simvastatin, which is in line with the fact that they are more electron-affinity and electrophilic. The fact that the MEP reactive sites align with the docking interaction residues also indicates the validity of the computational predictions. It is interesting to note that the electrostatic distribution of FHHA2 is more delocalized, which predisposes it to a variety of enzyme interactions and in line with the higher docking affinity and greater lipid-lowering potential. Conversely, FHHA1 exhibits more localized high density around the hydrazone nitrogen, which is associated with higher hydrogen-bond donating potential and which is linked with its lower IC_50_ value in the DPPH antioxidant experiment.

**Fig. 11:**
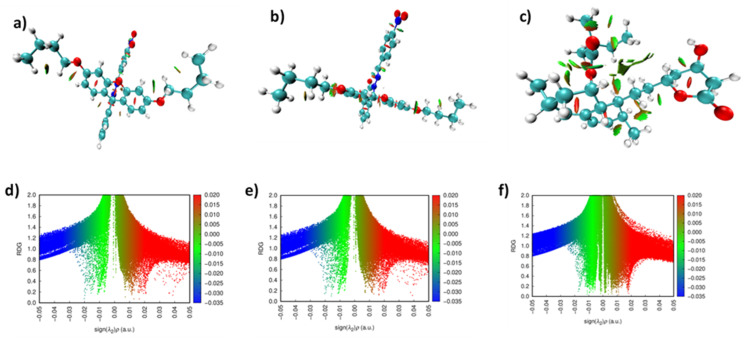
NCI surface of (a) FHHA1 (b) FHHA2 (c) simvastatin. RDG scatter plot of (d) FHHA1 (e) FHHA2 (f) simvastatin.

The NCI surfaces and RDG scatter plots (Fig. 11d–f), which illustrate weak non-covalent interactions in the molecular structures. In FHHA1 (Fig. 11a and d), isosurfaces of green color are concentrated over aromatic and heteroatomic regions, which means that there is a van der Waals interaction to stabilize the hydrophobic enzyme pockets, whereas red and blue surfaces are not well represented, which reflects the presence of minor steric repulsions and attractive forces, respectively.^75^ FHHA2 (Fig. 11b and e) has larger and continuous green surfaces with stronger van der Waals stabilization and higher intramolecular flexibility with RDG plots showing pronounced spikes at the negative *λ*_2_*ρ* region.

However, the converse is observed with simvastatin (Fig. 11c and f) as the density is near-zero and the negative *λ*_2_*ρ* spikes are smaller, indicating weaker repulsive dispersive interactions and weak steric repulsion, which restrict the conformational flexibility.^76^ In general, FHHA2 has the best balance between the appealing and repulsive interactions, which is in line with the lower hardness, higher softness, and greater electrophilicity of the material based on DFT measures. This quantum-level interaction profile is not only consistent with its high docking affinity (−8.5 kcal mol^−1^), but also consistent with experimental measurements, which indicate its superior stabilization in biological systems and increased activity in lipid-lowering action, although FHHA1 local interactions are consistent with its specialized antioxidant activity.

The combined computational results provide a unified mechanistic framework that quantitatively explains the experimentally observed SAR trade-off (Table 4). The *para*-nitro substitution in FHHA2 optimizes docking affinity (−8.5 kcal mol^−1^), electrophilicity (*ω* = 5.05 eV), and polar interactions (Tyr517, Gln814), correlating with its broadest antihyperlipidemic activity (TC↓, TG↓, LDL-c↓). Conversely, the *meta*-nitro in FHHA1 preserves localized HOMO electron density on CH=N and moderated electrophilicity (*ω* = 4.45 eV), explaining its superior antioxidant activity (IC_50_ = 140.64 µg mL^−1^) and selective LDL-c reduction.

**Table 4:** Integrated correlation between computational descriptors and experimental biological activities

Compound	NO_2_ position	Docking score (kcal mol^−1^)	*ω* (eV)	Experimental antihyperlipidemic	Experimental antioxidant IC_50_ (µg mL^−1^)
FHHA1	*meta*	−8.1	4.45	Selective LDL-c↓	140.64 (most active)
FHHA2	*para*	−8.5	5.05	TC↓, TG↓, LDL-c↓ (broadest)	181.91
Simvastatin	—	−7.7	1.71	Reference standard	N/A

This computational-experimental correlation validates the nitro group positional isomerism as the critical molecular determinant of the dual pharmacological profile. FHHA2 emerges as the lead candidate for antihyperlipidemic drug development due to its optimal electronic properties and superior enzyme binding, directly matching its experimentally demonstrated broad-spectrum lipid reduction. FHHA1 represents a specialized antioxidant scaffold with preserved hydrogen-donating capacity, consistent with its lower IC_50_ in radical scavenging assays.

#### 3.3.4. Molecular dynamics simulation

To further confirm the docking outcome and study the dynamics stability of the ligand–protein complexes in physiological conditions, molecular dynamics (MD) simulations were performed over 100 ns in the GROMACS simulation package. The simulations have been conducted in case of HMG-CoA reductase complexes of FHHA1 and FHHA2 in addition to reference drug simvastatin. Root mean square deviation (RMSD), root mean square fluctuation (RMSF), radius of gyration (*R*_g_), solvent accessible surface area (SASA), hydrogen bond analysis and density distribution were used to examine the structural stability and conformational dynamics. These parameters all give information about structural deviation, residue flexibility, compactness, solvent exposure, intermolecular stabilization, system equilibrium at the simulation trajectory.

**Fig. 12:**
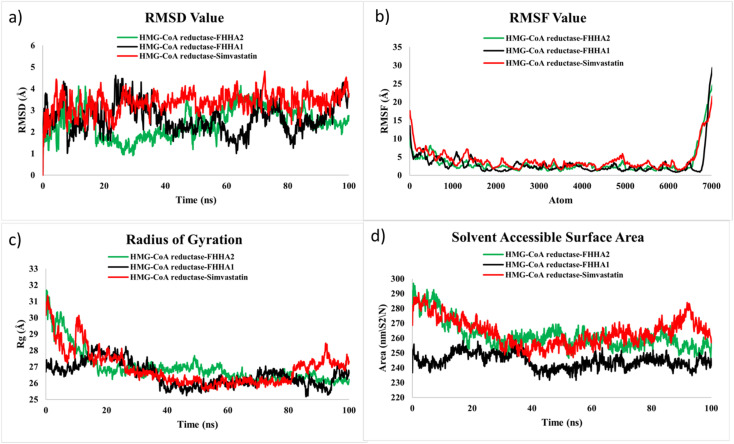
(a) RMSD, (b) RMSF, (c) RG and (d) SASA of FHHA1, FHHA2, Simvastin and HMG-CoA reductase complex.

To assess the structural stability of the complexes in general, the root mean square deviation (RMSD) analysis of the backbone atoms (Fig. 12a) was conducted. Upon an initial equilibration step, the HMG-CoA reductase-FHHA2 complex stabilized at an average of about 2.0–2.8 Å with a fairly small range of variations during the 100 ns simulation. Comparatively, the FHHA1 complex had some marginally more fluctuations of as much as 3.0–3.5Å whereas the simvastatin complex had the most extreme deviations of up to 4.0 Å at a few points. The lower and more consistent values of RMSD of FHHA2 suggest a greater conformational stability in the enzyme catalytic pocket. This is in line with the docking outcome, as FHHA2 had the best binding affinity (−8.5 kcal mol^−1^), and numerous polar and hydrophobic interactions with important residues including Tyr517, Gln814, and Ile536. The *para*-nitro replacement in the FHHA2 structure facilitates positive electronic delocalization and geometric orientation of the ligand, which enables it to be firmly stabilized in the active site in dynamic movement.

RMSF analysis was conducted in order to further explore the flexibility of residue levels (Fig. 12b). The residues of the FHHA2 complex had comparatively low fluctuations in most of them as compared to FHHA1 and simvastatin, which showed that the backbone was not very mobile when bound to a ligand. The biggest dynamics were in the N- and C-terminal regions, which are generally more free as they are exposed to the solvent. Notably, the residues which were found around the catalytic region exhibited lesser changes of the FHHA2 complex which indicates that the FHHA2 binds to the binding pocket better. The enhanced stabilization is probably due to the extra polar interactions that are enhanced by the *para*-nitro substituted aromatic system of FHHA2 that enhances intermolecular contacts and restrains the excessive movement of residues.

The radius of gyration (*R*_g_) parameter was used to determine the compactness of the protein structure during the simulation (Fig. 12c). The FHHA2 complex could retain a relatively constant value of *R*_g_ of about 2627 Å over the simulation period after the initial equilibration phase which implies that the compact protein conformation was retained. FHHA1 complex maintained a little higher changes in *R*_g_ but the simvastatin complex had relatively larger changes, which indicated more rearrangements in its conformational changes. The enhanced compactness seen in the FHHA2 complex indicates greater structural stabilization in the protein matrix, which is in line with the increased interaction network in the protein in docking analysis.

To determine the level of protein surface exposure to solvent molecules, solvent accessible surface area (SASA) analysis (Fig. 12d) was carried out. The FHHA2 complex had medium SASA values having relatively smooth changes throughout the entire trajectory, indicating stable folding and constant solvent accessibility. Conversely, the FHHA1 and simvastatin complexes had a little more significant changes in SASA, meaning that there were more structural changes that occurred in the course of the simulation. The balanced SASA profile of FHHA2 suggests the accommodation in the hydrophobic binding cavity of HMG-CoA reductase and preserving the preferred solvent interactions.

**Fig. 13:**
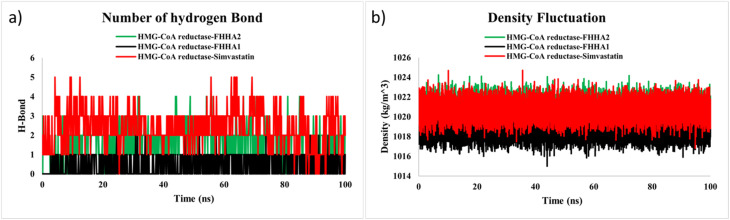
(a) Hydrogen bonding profile (b) density profile of FHHA1, FHHA2, Simvastin and HMG-CoA reductase complex.

The simulation was also used to monitor hydrogen bonding between the ligand and protein to determine the persistence of polar contacts (Fig. 13a). The FHHA2 complex always retained around 13 hydrogen bonds during the trajectory with intermittent highs of four interactions. These hydrogen bonds have been found to be comparatively stable throughout the simulation, which means that FHHA2 was stabilized in the catalytic pocket. This finding is consistent with the docking analysis in which FHHA2 hydrogen bonded with residues including Tyr517 and Gln814 as well as hydrophobically interacting with Ile536 and Ile762. The *para*-nitro group of FHHA2 presents the appropriate oxygen atoms with the right number of electrons that can serve as acceptors in hydrogen bonds and enable stable polar interaction that helps stabilize the complexes.

Conversely, the FHHA1 complex had lower numbers of hydrogen bonds, which typically varied between 0 and 2 interactions throughout the simulation. The reduced hydrogen bonding frequency indicates a relatively weak stabilization in the binding pocket. This disparity could be explained by the *meta*-nitro replacement in FHHA1 that limits the optimal spatial orientation to hydrogen bond with active-site residues. As a result, FHHA1 is more stabilized by hydrophobic interactions, and this is the reason why its RMSD and RMSF swings are larger.

The simvastatin complex exhibited more hydrogen bonds, which were usually varying between 2 and 5 associations. Most of these interactions though were not persistent, but temporary meaning that the trajectory underwent many rearrangements. This oscillatory action justifies the greater RMSD fluctuations of the simvastatin complex even though there were many hydrogen bonds.

To assess the thermodynamic stability of the solvated simulation systems, the density fluctuation analysis (Fig. 13b) was conducted. The density profile of the FHHA2 complex was stable with a centred value of around 1022 kg m^−3^ with minimal variation, which showed that the system was in equilibrium and in good balance during the simulation. The simvastatin system exhibited the same density values with a little broader variation, whereas the FHHA1 system exhibited slightly lower density values (∼1017 1018 kg m^−1^). Though all systems were within the acceptable density ranges to perform a stable simulation, the FHHA2 system showed the most consistent system density profile indicating greater efficiency in packing and stability of the system.

The molecular dynamics (MD) simulations findings support the results of the docking and density functional theory (DFT), where FHHA2 is the most stable ligand out of the compounds studied. FHHA2 had significantly lower RMSD, less residue variation in RMSF analysis, and greater structural compactness in terms of radius of gyration (*R*_g_) values. In addition, its balanced solvent-accessible surface area (SASA), maintained density profile, and maintained hydrogen bonding interaction reveals high levels of conformational stability during the simulation.

These dynamic characteristics are in line with its positive electronic characteristics based on DFT computations, such as lower energy gap, higher electrophilicity, and higher molecular softness, which enables successful charge–transfer reactions with catalytic residues. Having a *para*-nitro substituent in FHHA2 also leads to the creation of a larger and more stable interaction network in the active site, especially with key residues including Tyr517 and Gln814, thus increasing electrostatic and hydrogen bonding interactions.

As a result, FHHA2 shows a better ligand retention and binding stability than FHHA1 and simvastatin. Altogether, the combined computational studies confirm that FHHA2 is the most stable and persistent complex with HMG-CoA reductase, which supports the idea that it is a promising antihyperlipidemic lead candidate among the synthesized spiro-lactam derivatives.

#### 3.3.5. Drug-likeness and ADME-Tox evaluation

The ADME-Tox profiling was performed to rationalize the experimentally observed safety profile and predict the pharmacokinetic behavior of FHHA1 and FHHA2, particularly in light of their LD_50_ > 1000 mg kg^−1^ demonstrated in acute toxicity studies and their dose-dependent efficacy at 40 mg kg^−1^ in antihyperlipidemic assays.

Drug-likeness evaluation reveals that FHHA1 and FHHA2 share identical physicochemical profiles, with molecular weight (563.60 g mol^−1^) and *i* log *P* (4.62) indicating strong lipophilicity and favorable membrane permeability. This high lipophilicity correlates with their demonstrated biological activity at 40 mg kg^−1^, as it facilitates intestinal absorption and cellular uptake necessary for enzyme inhibition and radical scavenging.^77^ Although their aqueous solubility (log *S* = −7.67) is lower than simvastatin (log *S* = −3.56), this characteristic is commonly observed in lipophilic antihyperlipidemic agents and does not preclude oral bioavailability, as evidenced by their *in vivo* efficacy after oral administration (Tables 5–7).

**Table 5:** ADMET analysis of FHHA1

		Post-docking analysis	
Compound	Name of analysis	Properties	Values
FHHA1	ADME analysis	Mass (g mol^−1^)	563.60
		*i*Log *p*	4.62
		H donor	0
		H acceptor	7
		Water solubility (mg ml^−1^)	1.21 × 10^−5^
		Rotational bonds	9
		TPSA (Å^2^)	106.18
		Molar refractivity	165.17
		Log *S*	−7.67
FHHA1	Toxicity profiling	Toxicity class	4
		LD_50_ (mg Kg^−1^)	1000
		Carcinogenicity	Active
		Mutagenicity	Inactive
		Cytotoxicity	Inactive
		Hepatotoxicity	Inactive
		Thyroid hormone receptor beta (THRβ)	Inactive
		Estrogen receptor alpha (ER)	Inactive
		Neurotoxicity	Inactive
		Cardiotoxicity	Inactive

**Table 6:** ADMET analysis of FHHA2

		Post-docking analysis	
Compound	Name of analysis	Properties	Values
Simvastatin	ADME analysis	Mass (g mol^−1^)	418.57
		*i* log *p*	3.74
		H donor	1
		H acceptor	5
		Water solubility (mg ml^−1^)	5.01 × 10^−3^
		Rotational bonds	72
		TPSA (Å^2^)	72.83
		Molar refractivity	118.47
		Log *S*	−3.56
Simvastatin	Toxicity profiling	Toxicity class	4
		LD_50_ (mg Kg^−1^)	1000
		Carcinogenicity	Active
		Mutagenicity	Inactive
		Cytotoxicity	Inactive
		Hepatotoxicity	Inactive
		Thyroid hormone receptor beta (THRβ)	Inactive
		Estrogen receptor alpha (ER)	Inactive
		Neurotoxicity	Inactive
		Cardiotoxicity	Inactive

**Table 7:** ADMET analysis of simvastatin

		Post-docking analysis	
Compound	Name of analysis	Properties	Values
FHHA2	ADME analysis	Mass (g mol^−1^)	563.60
		*i* log *p*	4.62
		H donor	0
		H acceptor	7
		Water solubility (mg ml^−1^)	1.21 × 10^−5^
		Rotational bonds	9
		TPSA (Å^2^)	106.18
		Molar refractivity	165.17
		Log *S*	−7.67
FHHA2	Toxicity profiling	Toxicity class	4
		LD_50_ (mg Kg^−1^)	1000
		Carcinogenicity	Active
		Mutagenicity	Inactive
		Cytotoxicity	Inactive
		Hepatotoxicity	Inactive
		Thyroid hormone receptor beta (THRβ)	Inactive
		Estrogen receptor alpha (ER)	Inactive
		Neurotoxicity	Inactive
		Cardiotoxicity	Inactive

The predicted pharmacokinetic and toxicity profiles support the experimental observations regarding the safety and biological activity of the synthesized compounds. The absence of major toxicity alerts for spiro-lactam derivatives and the LD_50_ value of 1000 mg kg^−1^ is consistent with their favorable tolerability observed in the biological evaluation. These findings further suggest that the spiro-lactam scaffold may represent a promising and safer alternative to the reference drug.

Collectively, docking, DFT, MEP and ADME-Tox results provide a consistent mechanistic picture of the observed activity trends. Although simvastatin has proven efficacy and positive pharmacokinetic characteristics, comparison of its energy gap and hardness indicates reduced electronic adaptability. Conversely, FHHA2 has high binding affinity, improved electronic softness, increased electrophilicity, and desirable electrostatic distribution, exhibiting a balanced stability and reactivity profile.

Hence, FHHA2 is the most promising lead compound of those evaluated. Its high docking affinity, optimum frontier molecular orbital properties and suitable reactivity descriptors indicate enhanced ability to stabilize interactions in the enzymatic binding pocket. These results should be supported by molecular dynamics simulation and extended experimental biological assessment to prove its therapeutic potential in antihyperlipidemic drug development.

## 4. Conclusion

The present study describes the synthesis of novel spiro-lactam hydrazone derivatives (FHHA1, FHHA2) using Es-sifa natural clay as a bifunctional heterogeneous base catalyst (condensation/alkylation without organic base). This three-step protocol achieves 86–87% overall yields in 36 min (*vs.* 34.1–37.9% in 68 h by conventional heating), representing a 113-fold acceleration with significant energy savings. Both compounds exhibit a good safety profile, with LD_50_ values ranging from 2000 to 5000 mg kg^−1^, FHHA1 selectively reduces LDL-c, while FHHA2 acts as a broad-spectrum lipid modulator with superior antioxidant activity. Computational studies validate their enhanced target engagement (binding energies −8.1/−8.5 kcal mol^−1^) and favourable electronic properties compared to simvastatin. These spiro-lactam hydrazones represent a promising, sustainable scaffold linking green synthesis and polypharmacology for metabolic disease treatment. Future work will focus on long-term toxicity evaluation and pharmacokinetic optimization to advance toward preclinical development.

## Safety and hazards

All chemical reagents were handled following standard laboratory safety protocols. Microwave-assisted reactions were conducted in sealed vessels with appropriate pressure control. Acute toxicity studies in rats were performed in compliance with institutional ethical guidelines. No exceptional hazards were identified beyond standard organic synthesis and animal testing procedures. Appropriate personal protective equipment (PPE) was used throughout the study.

## Conflicts of interest

Authors declare no conflict of interest.

## Supplementary Material

RA-016-D6RA02313D-s001

## Data Availability

All additional data analyzed during this study can be found in the supplementary information (SI) section of this article. Supplementary information: ^1^H and ^13^C NMR, HRMS, and IR spectra, as well as the complete spectral data for all synthesized compounds. See DOI: https://doi.org/10.1039/d6ra02313d.
