# Supplementary material for: Synthesis of spiro-lactam hydrazones by clay catalysis: toxicity, antioxidant, hypolipidemic and In silico assessments
Source: RSC Adv. 2026 May 5;16(25):22949–68. doi: 10.1039/d6ra02313d (PMC13142659; doi:10.1039/d6ra02313d)
Supplement: RA-016-D6RA02313D-s001 [file RA-016-D6RA02313D-s001.pdf]

Supplementary Information (SI) for RSC Advances.
This journal is © The Royal Society of Chemistry 2026

Synthesis of Spiro-Lactam Hydrazones by Clay Catalysis: Toxicity, Antioxidant, Hypolipidemic and *In-silico* assessments

Mohammed El Mesky[1]*, Hicham Zgueni [1], Ismail bouadid [2], Jarin Tasnim[3], Yassine Rhazi[4], Md Mehedi Hasan[3], Tanghourte Mohamed[1], Adil Qabouche[2], Mohammed Chalkha[1], Na'il Saleh[5], Driss Chebabe[1], El Houssine Mabrouk[1,4] and Mohamed Eddouks[2]

[1]*Laboratory of Materials Engineering for the Environment and Natural Resources, Faculty of Sciences and Techniques, University of Moulay Ismail of Meknes, B.P 509, Boutalamine, 52000, Errachidia, Morocco.*

[2]*Team of Ethnopharmacology and Pharmacognosy, Faculty of Sciences and Techniques Errachidia, Moulay Ismail University of Meknes, Errachidia, Morocco*

[5]*Department of applied chemistry and chemical Engineering, University of rajshahi, rajshahi Bangladesh.*

[4]*Laboratory of Engineering of Organometallic, Molecular Materials, Environment, and Innovative Pedagogy (LIMOMEPI), Faculty of Sciences Dhar EL Mahraz, Sidi Mohamed Ben Abdellah University, P.O. Box 1796 (Atlas), 30000 Fez, Morocco.*

[5] *Chemistry Department, College of Science, United Arab Emirates University P.O.Box 15551, Al Ain, United Arab Emirates*

***Corresponding authors' E-mails**: *El MESKY Mohammed m.elmesky@edu.umi.ac.ma*

I. Chemical reagents and instruments

All chemicals, solvents and reagents used were of analytical grade and used without further purification. The chemicals were purchased from commercial suppliers: Fluorescein (98.0 %), hydrazine (99.0 %), Ethanol (≥99.5 %), (99.8%), 3-Nitrobenzaldhyde (98.0 %),4-nitrobenzaldhyde (98.0 %), Bromobutan (99.8%), ACN (99.8 %), Et_3N (≥98.0 %), (≥98.0 %),, Hexane (≥95%), Acetate d'ethyle (≥98%%).

1. *NMR ^1H, ^{13}C and HRMS Spectra of FH*

2-amino-3',6'-dihydroxyspiro[isoindolin-3-one-1,9'-xanthene] FH *had a yield of Yield = 96.0%, a light off-white solid. f.r = 0.2 (hexane/ether) (2/1).*

^{1}H NMR (300 MHz, DMSO-d6). δ (ppm) = 9.84 (s, 2H, NH_2 hydrazide), 9.02 (s, 1H, OH), 7.91–7.85 (m, 1H, ArH), 7.58 (dtd, J=21.4, 7.4, 1.2 Hz, 2H, ArH), 7.38 (dd, J=6.8, 3.0 Hz, 2H,ArH), 7.31 (dd, J=5.1, 1.9 Hz, 3H, ArH), 7.10 (dd, J=7.4, 1.2 Hz, 1H, ArH), 6.62 (d, J=2.3 Hz, 2H, ArH), 6.46 (d, J=8.6 Hz, 2H, ArH), 6.42 (dd, J=8.6, 2.4 Hz, 2H, ArH).

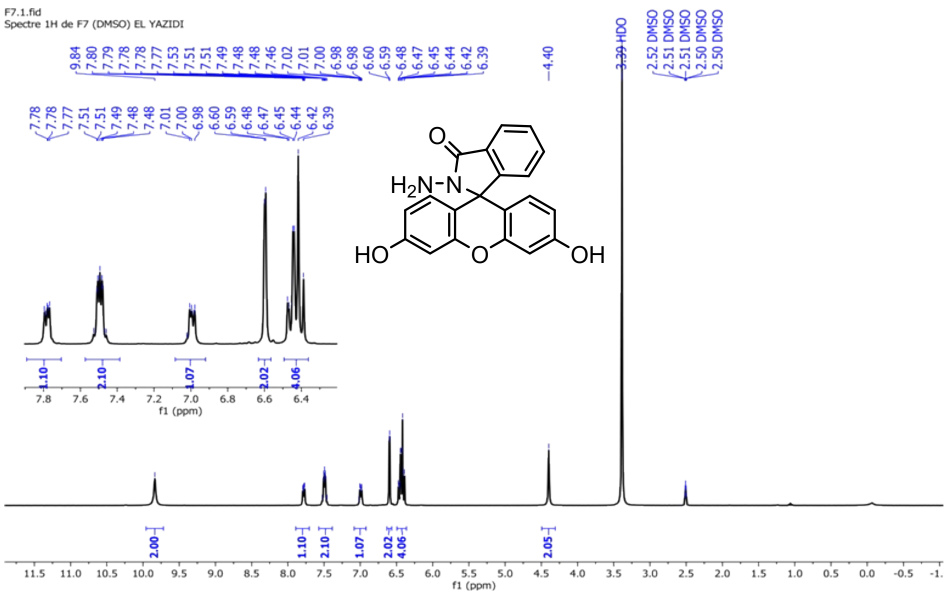

Figure S 1. 1H NMR spectrum (300 MHz, DMSO-d6) of FH

^{13}C NMR (126 MHz, DMSO-d6): δ (ppm) = 164.17 (C=O), 159.13, 152.81, 150.91, 149.80, 135.04, 134.52, 130.89, 129.66, 129.65, 129.36, 128.54, 127.29, 124.36, 123.73, 112.85, 110.75, 103.01 (aromatic carbons), 65.93 (spiro carbon);

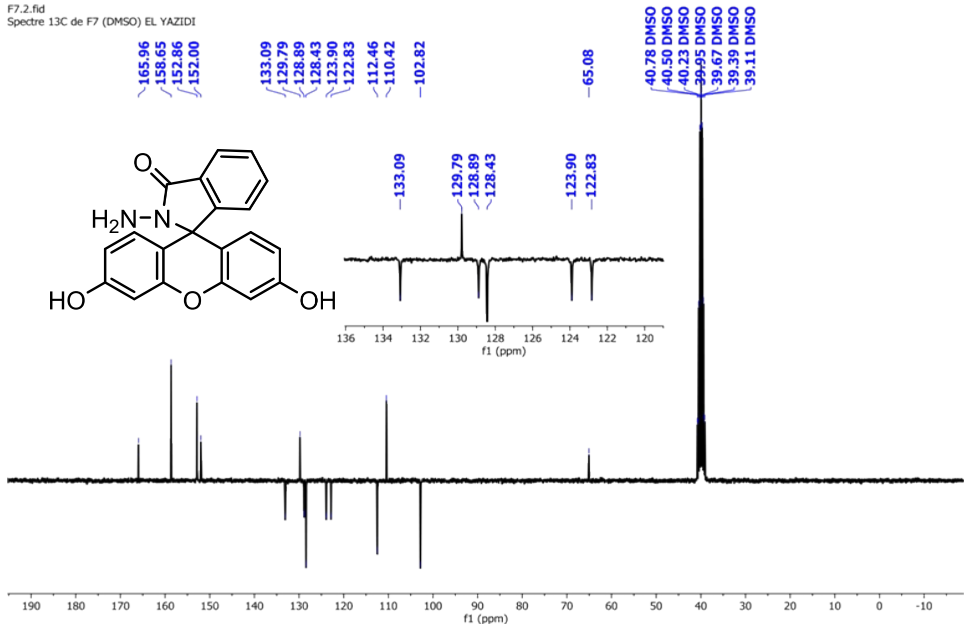

Figure S 2. ^{13}C NMR spectrum (75MHz, DMSO-d6) of FH

HRMS (m/z). Calculated for $C_{20}H_{14}N_2O_4$ 346.10, found 345.08569, Δm = -0.01431 Da

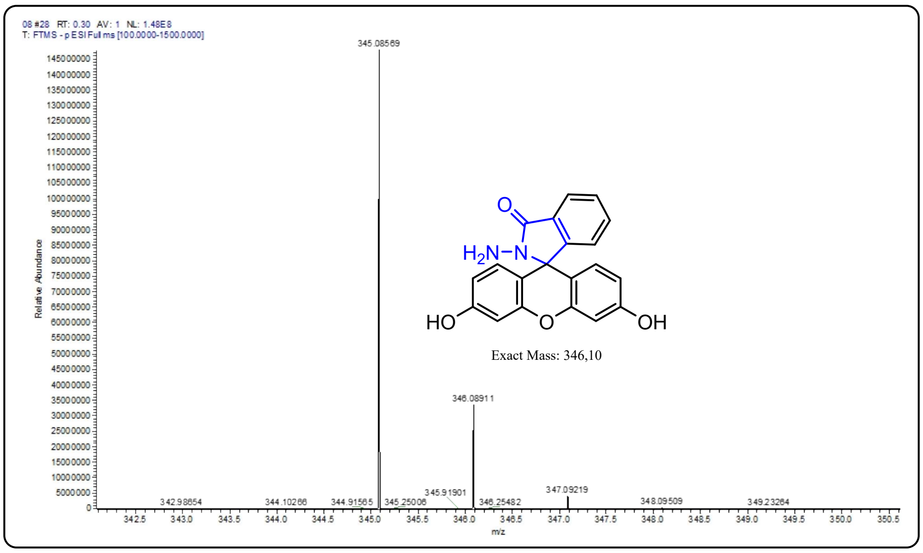

Figure S 3. Mass spectrum of FH

1. ***NMR ^{1}H, ^{13}C and HRMS Spectra of FHH1***

2-((3-Nitrobenzylidene)amino)-3',6'-dihydroxyspiro[isoindolin-3-one-1,9'-xanthene] FHH2 ***FHH1****: had a yield of Yield = 94.3%; Melting point: 197-199 °C, yellow. f.r (Ac: Hex)(3:1)= 0.68*

^{1}H NMR (500 MHz, DMSO-d 6). δ 9.87 (s, 2H), 9.13 (s, 1H), 8.22 (t, J = 2.0 Hz, 1H), 8.13 (dd, J = 8.0, 2.4 Hz, 1H), 7.91 (d, J = 7.5 Hz, 1H), 7.81 (d, J = 7.9 Hz, 1H), 7.67 – 7.54 (m, 3H), 7.13 (d, J = 7.5 Hz, 1H), 6.65 (d, J = 2.3 Hz, 2H), 6.50 – 6.39 (m, 4H).

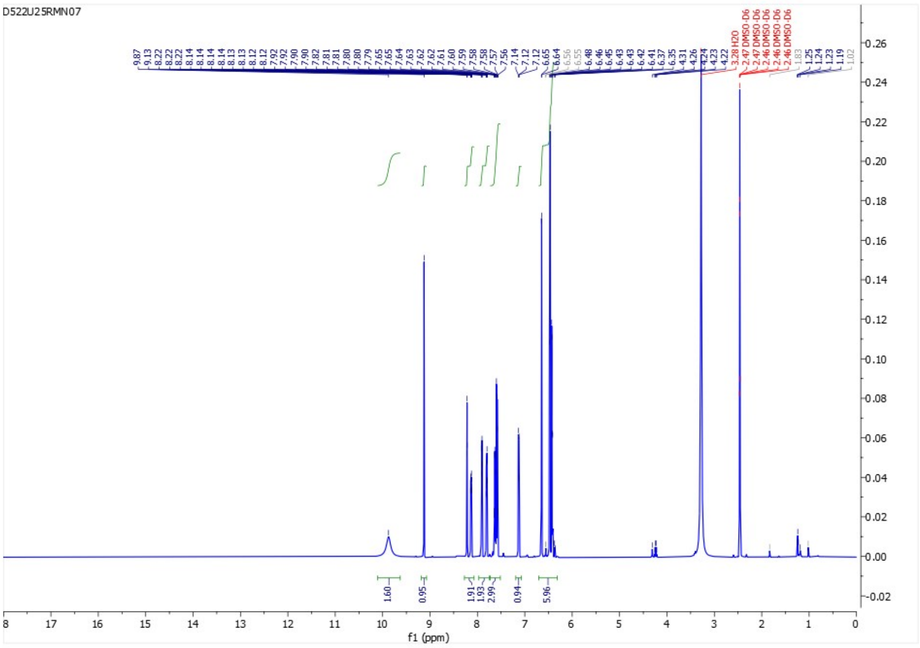

Figure S 4. 1H NMR spectrum (500 MHz, DMSO-d6) of FHH1

^{13}C NMR (126 MHz, DMSO-d 6). δ 164.40, 159.23, 152.89, 150.81, 148.77, 146.86, 136.92, 134.87, 133.41, 131.00, 129.80, 129.28, 128.45, 124.98, 124.46, 123.93, 121.24, 112.93,110.57, 103.10, 66.11.

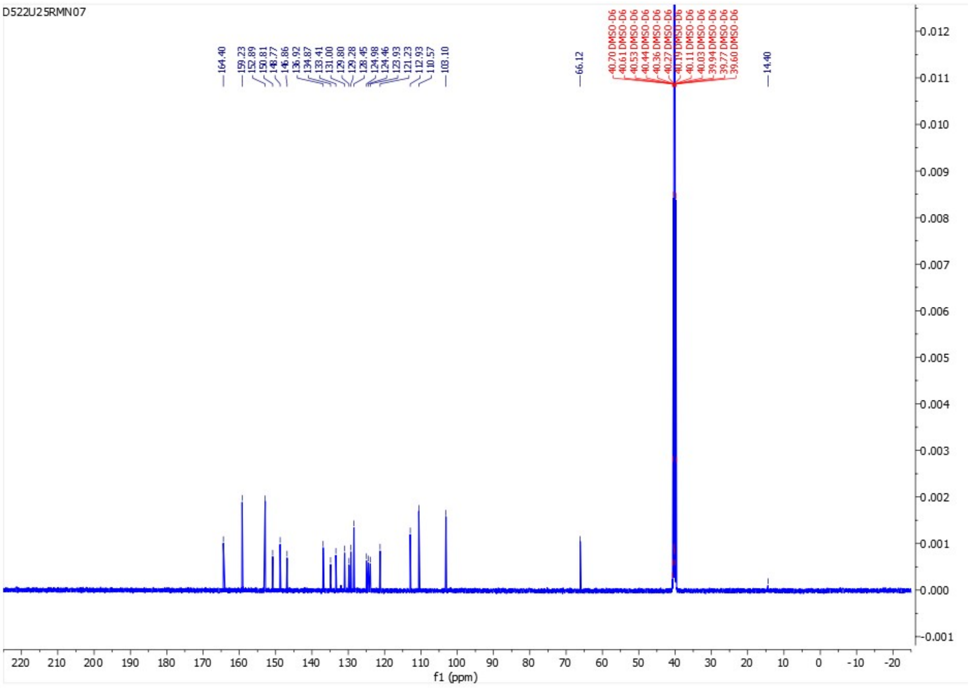

Figure S 5. ^{13}C NMR spectrum (125 MHz, DMSO-d6) of FHH1

1. ***NMR ^{1}H, ^{13}C and HRMS Spectra of FHH2***

2-((4-Nitrobenzylidene)amino)-3',6'-dihydroxyspiro[isoindolin-3-one-1,9'-xanthene] FHH2 had a yield of 94.5%; M.p: 180-182 °C, yellow. f.r. (Ac:Hex)(3:1) = 0.67;

^{1}H NMR (500 MHz, DMSO-d6 δ 10.02 (d, J = 112.9 Hz, 2H), 9.11 (s, 1H), 8.16 (d, J = 8.6 Hz, 2H), 7.91 (d, J = 7.5 Hz, 1H), 7.64 (dd, J = 7.9, 5.2 Hz, 3H), 7.58 (t, J = 7.4 Hz, 1H), 7.12(d, J = 7.6 Hz, 1H), 6.64 (d, J = 2.3 Hz, 2H), 6.47 (d, J = 8.6 Hz, 2H), 6.42 (dd, J = 8.7, 2.4Hz, 2H).

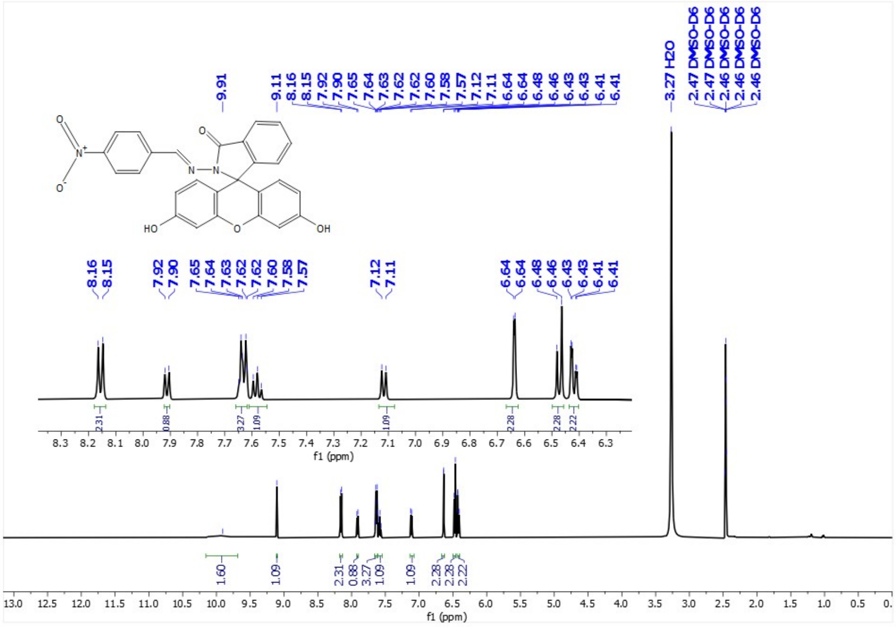

Figure S 6. ^{1}H NMR spectrum (500 MHz, DMSO-d6) of FHH1

^{13}C NMR (500 MHz, DMSO-d6) δ 164.56, 159.27, 152.77, 150.98, 148.54, 146.54, 141.20, 134.99, 129.82, 129.09, 128.50, 128.14, 124.65, 124.47, 123.97, 112.99, 110.44, 103.11, 66.14.;

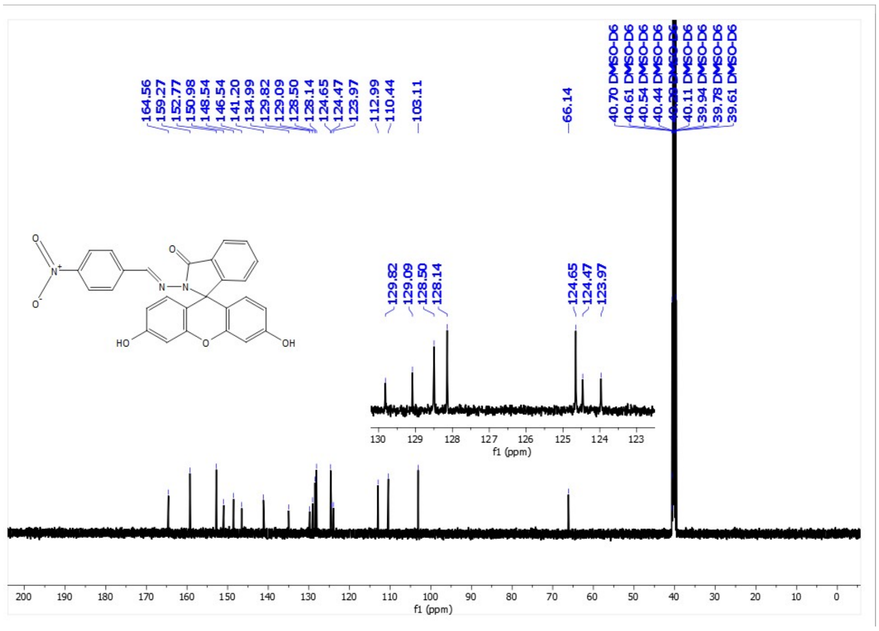

Figure S 7. ^{13}C NMR spectrum (125 MHz, DMSO-d6) of FHH1

HRMS (m/z). Calculated for C27H17N3O6 480.11174, found 480.11212, Δm = 1.002 Da.

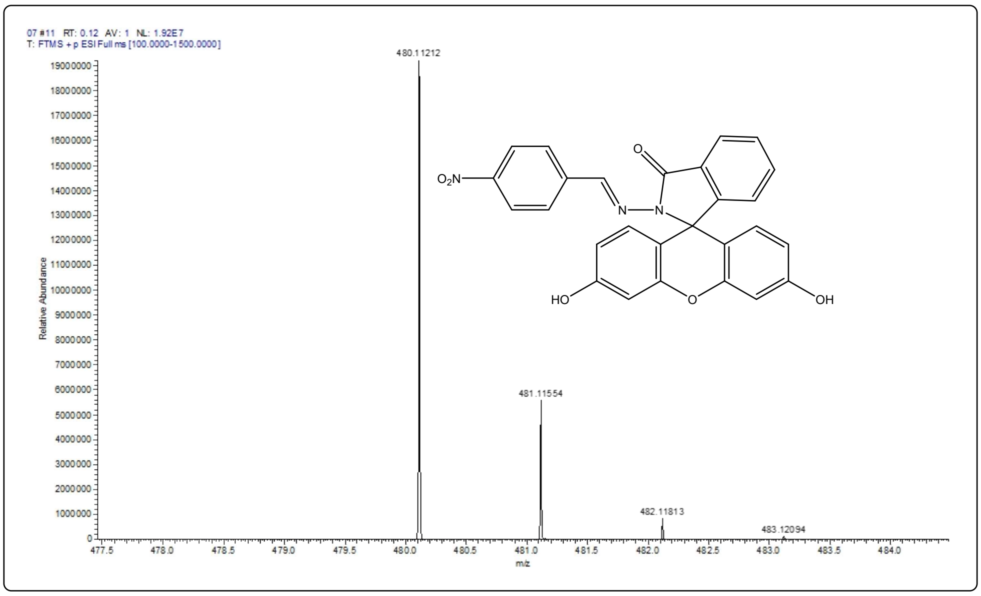

Figure S 8. Mass spectrum of FH

1. ***NMR ^{1}H, ^{13}C and HRMS Spectra of FHHA1***

3',6'-Dibutoxy-2-((3-nitrobenzylidene)amino)spiro[isoindolin-3-one-1,9'-xanthene] *FHHA1, had a yield of 95.0%; M.p: 129-131 °C, yellow. f.r. (Ac:Hex)(4:1) = 0.63.*

^{1}H NMR (500 MHz, DMSO-d6). δ 9.24 (s, 1H), 8.21 (t, J = 1.9 Hz, 1H), 8.14 (ddd, J = 8.1, 2.4, 1.0 Hz, 1H), 7.96–7.90 (m, 1H), 7.81 (dt, J = 7.8, 1.3 Hz, 1H), 7.69–7.57 (m, 3H), 7.14 (dt, J = 7.6, 0.9 Hz, 1H), 6.85 (dd, J = 2.1, 0.8 Hz, 2H), 6.62 – 6.55 (m, 4H), 3.95 (t, J = 6.5 Hz, 4H), 1.67–1.60 (m, 4H), 1.37 (h, J = 7.4 Hz, 4H), 0.87 (t, J = 7.4 Hz, 6H).

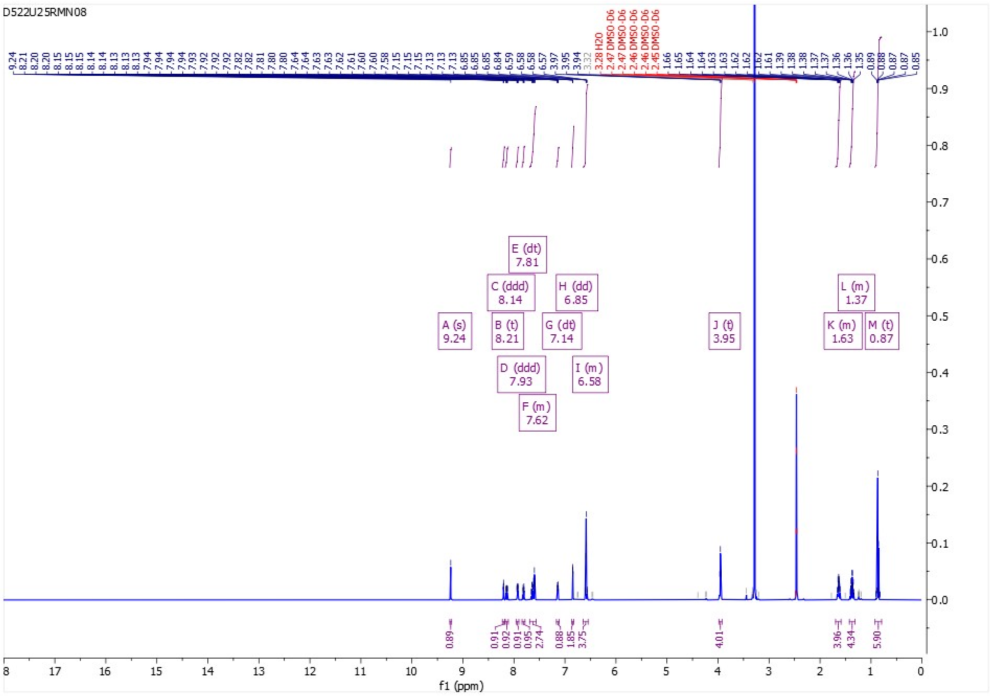

Figure S 9. ^{1}H NMR spectrum (500 MHz, DMSO-d6) of FHHA1

^{13}C NMR (126 MHz, DMSO-d6). δ 164.53, 160.37, 152.9, 150.52, 148.75, 147.62, 134.97, 133.42, 131.01, 130.00, 128.50, 125.09, 124.48, 124.06, 121.34, 112.48, 111.92, 102.04, 68.13, 66.00, 31.15, 19.22, 14.12.

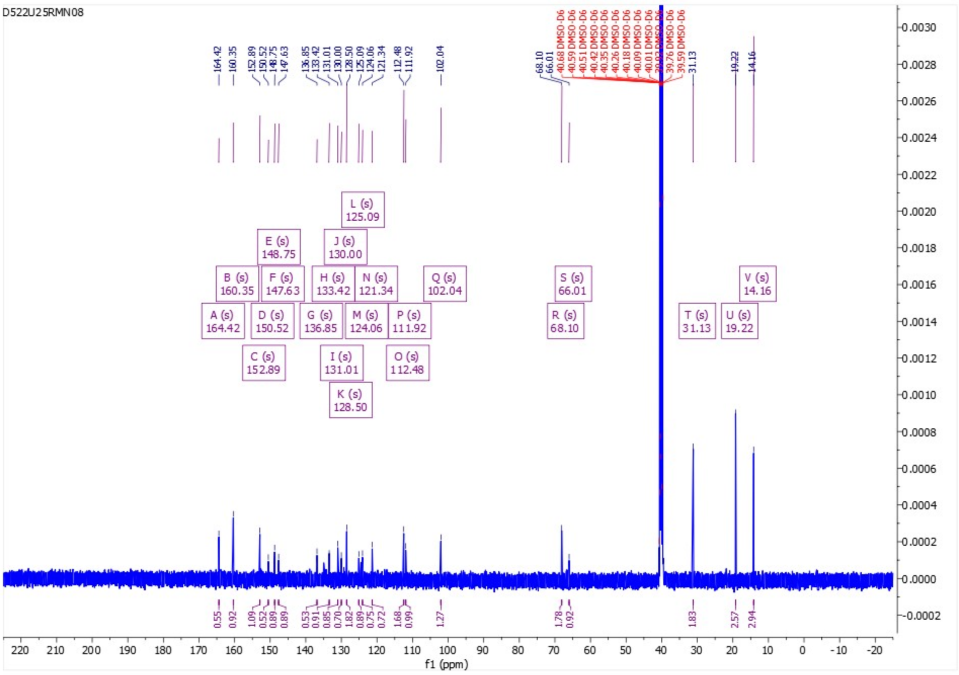

Figure S 10. ^{13}C NMR spectrum (125 MHz, DMSO-d6) of FHHA1

HRMS (m/z). Calculated for $C_{35}H_{33}N_3O_6$ 592.23694, found 592.23609, Δm = 0.996 Da.

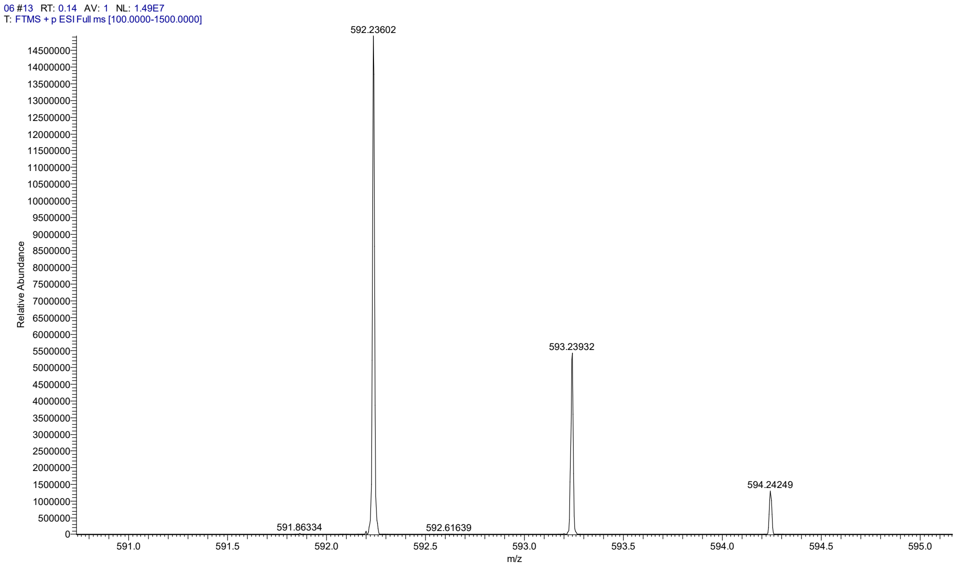

Figure S 11. Mass spectrum of FHHA1

1. ***NMR ^{1}H, ^{13}C HRMS and IR Spectra of FHHA2***

3',6'-Dibutoxy-2-((4-nitrobenzylidene)amino)spiro[isoindolin-3-one-1,9'-xanthene] ***FHHA2,*** *had a yield of 96.0%; M.p: 122–124 ^{0}C, yellow. f.r. (Ac:Hex)(4:1) = 0.65.*

¹H NMR (500 MHz, DMSO-d6). δ 9.32 (s, 1H), 7.95–7.88 (m, 2H), 7.69–7.51 (m, 5H), 7.09 (dt, J = 7.5, 1.0 Hz, 1H), 6.83 (t, J = 1.4 Hz, 2H), 6.59 (d, J = 1.4 Hz, 4H), 3.95 (t, J = 6.5 Hz, 4H), 1.68–1.59 (m, 4H), 1.43–1.31 (m, 4H), 0.87 (t, J = 7.4 Hz, 6H).

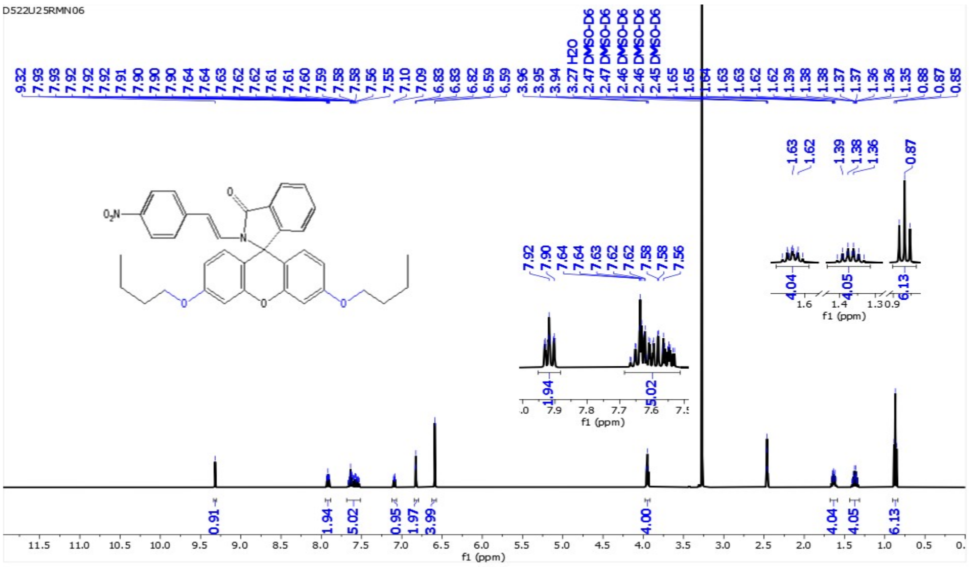

Figure S 12. ^{1}H NMR spectrum (500 MHz, DMSO-d6) of FHHA2

¹³C NMR (126 MHz, DMSO-d6). δ 164.72, 160.39, 152.64, 148.64, 143.56, 135.08, 134.19, 131.46, 129.93, 129.12, 128.72, 128.43, 127.59, 125.18, 124.38, 124.07, 112.56, 111.51, 102.17, 68.12, 65.76, 31.11, 19.21, 14.15.

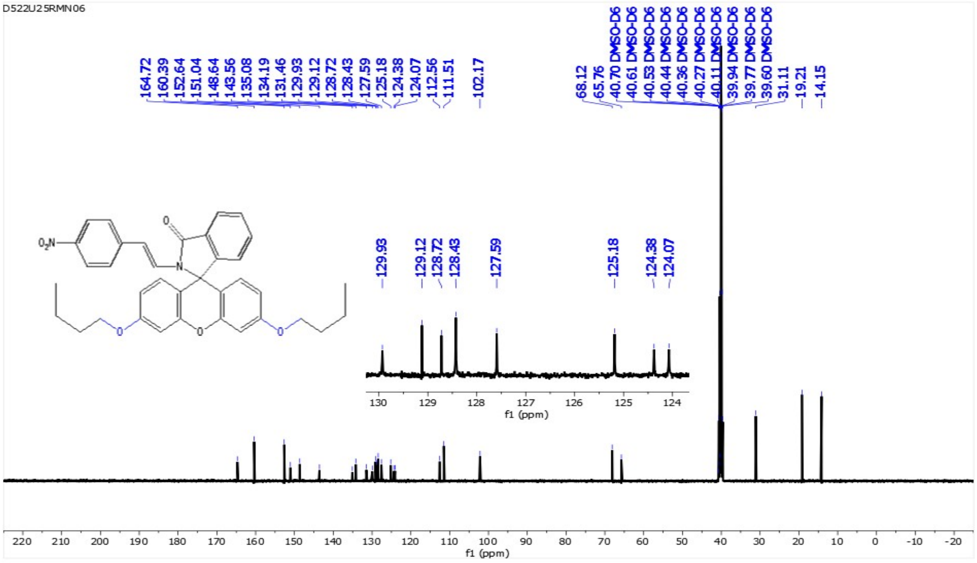

Figure S 13. ^{13}C NMR spectrum (125 MHz, DMSO-d6) of FHHA2

HRMS (m/z). Calculated for $C_{35}H_{33}N_3O_6$ 592. 23694, found 592.23609, Δm = 0.996 Da.

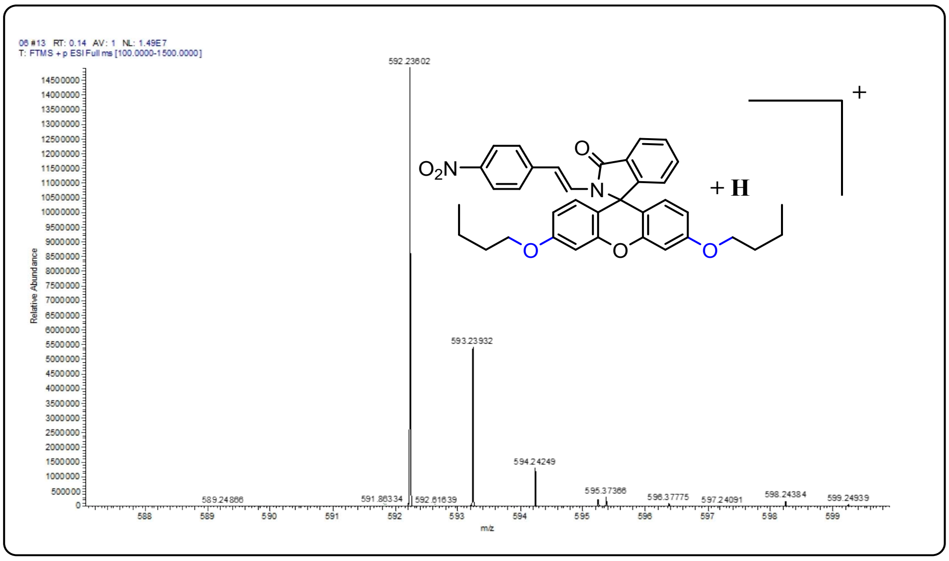

Figure S 14. Mass spectrum of FHHA2

IR ṽ (cm⁻¹): 2989–2872 (νCH₂, νCH₃); 3089–3020 (νC–H aromatic); 1702 (νC=O lactam/ester); 1614–1580 (νC=C aromatic); 1415 (ν NO₂); 1344 (νC–N); 1301–1189 (ν C–O–C ether); 1108–1014 (νC–O, νC–N); 873–684 (δC–H aromatic).

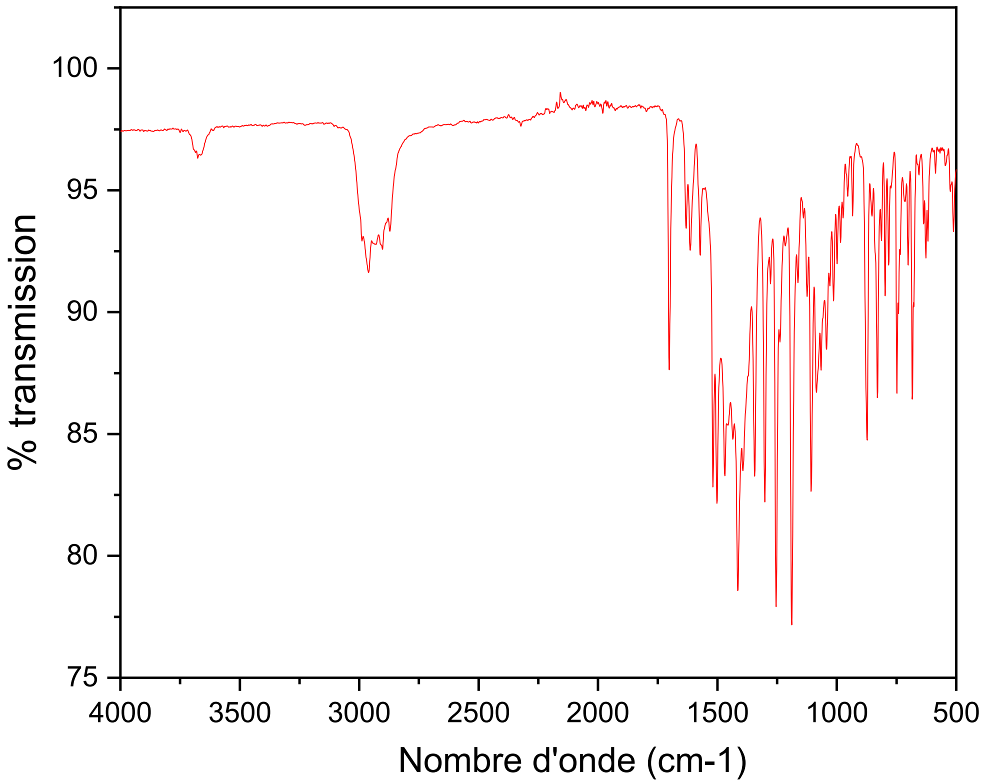

Figure S 15. IR spectrum of FHHA2
